# Old World Arenaviruses Enter the Host Cell via the Multivesicular Body and Depend on the Endosomal Sorting Complex Required for Transport

**DOI:** 10.1371/journal.ppat.1002232

**Published:** 2011-09-08

**Authors:** Giulia Pasqual, Jillian M. Rojek, Mark Masin, Jean-Yves Chatton, Stefan Kunz

**Affiliations:** 1 Institute of Microbiology, University Hospital Center and University of Lausanne, Lausanne, Switzerland; 2 Genomics Institute of the Novartis Research Foundation, La Jolla, California, United States of America; 3 Department of Cell Biology and Morphology and Cellular Imaging Facility, University of Lausanne, Lausanne, Switzerland; Harvard Medical School, United States of America

## Abstract

The highly pathogenic Old World arenavirus Lassa virus (LASV) and the prototypic arenavirus lymphocytic choriomeningitis virus (LCMV) use α-dystroglycan as a cellular receptor and enter the host cell by an unusual endocytotic pathway independent of clathrin, caveolin, dynamin, and actin. Upon internalization, the viruses are delivered to acidified endosomes in a Rab5-independent manner bypassing classical routes of incoming vesicular trafficking. Here we sought to identify cellular factors involved in the unusual and largely unknown entry pathway of LASV and LCMV. Cell entry of LASV and LCMV required microtubular transport to late endosomes, consistent with the low fusion pH of the viral envelope glycoproteins. Productive infection with recombinant LCMV expressing LASV envelope glycoprotein (rLCMV-LASVGP) and LCMV depended on phosphatidyl inositol 3-kinase (PI3K) as well as lysobisphosphatidic acid (LBPA), an unusual phospholipid that is involved in the formation of intraluminal vesicles (ILV) of the multivesicular body (MVB) of the late endosome. We provide evidence for a role of the endosomal sorting complex required for transport (ESCRT) in LASV and LCMV cell entry, in particular the ESCRT components Hrs, Tsg101, Vps22, and Vps24, as well as the ESCRT-associated ATPase Vps4 involved in fission of ILV. Productive infection with rLCMV-LASVGP and LCMV also critically depended on the ESCRT-associated protein Alix, which is implicated in membrane dynamics of the MVB/late endosomes. Our study identifies crucial cellular factors implicated in Old World arenavirus cell entry and indicates that LASV and LCMV invade the host cell passing via the MVB/late endosome. Our data further suggest that the virus-receptor complexes undergo sorting into ILV of the MVB mediated by the ESCRT, possibly using a pathway that may be linked to the cellular trafficking and degradation of the cellular receptor.

## Author Summary

Old World arenaviruses include the prototypic lymphocytic choriomeningitis virus (LCMV) and the highly pathogenic Lassa virus (LASV) that causes a severe hemorrhagic fever in humans and infects several thousand individuals per year in Western Africa. Cell entry of a virus is the first step of every virus infection and represents a promising target for therapeutic intervention. We and others had shown that LCMV and LASV attach to a cellular receptor, α-dystroglycan, followed by internalization by endocytosis via a novel and unusual pathway. Here we investigated the largely unknown molecular mechanisms of cell entry of LASV and LCMV with the goal to identify host cell factors involved. We discovered that during cell entry LASV and LCMV pass through a particular intracellular compartment, the multivesicular body (MVB)/late endosome, which is implicated in the internalization and degradation of cellular membrane receptors. Productive infection of LASV and LCMV critically depended on cellular factors involved in the membrane dynamics and sorting processes in the MVB. Based on our studies, we propose a model for Old World arenavirus entry in which the viruses hijack a pathway that may be linked to the cellular trafficking and degradation of their cellular receptor.

## Introduction

Over the past decades, several arenaviruses have emerged as causative agents of severe viral hemorrhagic fevers (VHF) that belong to the most devastating human diseases [1]. The Old World arenavirus Lassa virus (LASV) is the most prevalent human pathogen among the arenaviruses, causing several hundred thousand infections per year in Africa with thousands of deaths [2], [3]. The fatality rate of Lassa fever in hospitalized patients is >15% [4], rising to more than 50% in some outbreaks [5]. There is currently neither an efficient cure nor an efficacious vaccine, making LASV arguably one of the most neglected tropical pathogens. The prototypic arenavirus lymphocytic choriomeningitis virus (LCMV) merits significant attention as a powerful tractable experimental model system to study virus-host interactions and also as a prevalent human pathogen of clinical significance in congenital infections [6], [7], [8]. Moreover, LCMV infection of immunosuppressed adults can result in severe disease and death [9], [10].

Arenaviruses are enveloped negative strand RNA viruses with a non-lytic life cycle. The genome of LASV consists of two single-stranded RNA species, a large segment encoding the virus polymerase (L) and a small zinc finger motif protein (Z), and a small segment encoding the virus nucleoprotein (NP) and glycoprotein precursor (GPC) [11]. GPC is processed into GP1, implicated in receptor binding, and the transmembrane GP2, which contains the viral fusion machinery, allowing fusion of the viral and the cellular membrane during viral entry.

Binding of a virus to its cellular receptor(s) and subsequent entry into target cells are the first steps of virus infection and a fundamental aspect of the virus-host cell interaction [12], [13]. The first cellular receptor for Old World arenaviruses was identified as α-dystroglycan (α-DG), the peripheral moiety of DG, a highly conserved and ubiquitously expressed cell surface receptor for extracellular matrix (ECM) proteins [14]. Initially encoded as a single polypeptide, DG is cleaved into the extracellular α-DG and membrane anchored β-DG [15]. DG is expressed in most cells of developing and adult tissues and provides a molecular link between the ECM and the actin-based cytoskeleton. Alpha-DG is a primary receptor for LASV, most isolates of LCMV, the African arenaviruses Mopeia and Mobala, as well as Clade C New World arenaviruses [14], [16].

Upon receptor binding, arenaviruses undergo endocytosis and are delivered to acidified endosomes where pH-dependent fusion between the viral and the cellular membrane occurs [17]. The pH optimum for LCMV and LASV fusion of <5.0 is remarkably low [18] suggesting fusion in a late endosomal compartment. Electron microscopic examination of LCMV entry demonstrated uptake of virions in smooth vesicles lacking a clathrin coat [17], [19]. LCMV and LASV enter cells predominantly via an unusual pathway of endocytosis that shows some dependence on membrane cholesterol [20], but is independent of clathrin and caveolin, and does not require dynamin, ARF6, flotillin, or actin [19], [20], [21]. Receptor-mediated endocytosis of LCMV and LASV occurs rapidly with pH-dependent fusion occurring after 16–20 minutes [19], [21], a kinetics similar to viral entry via clathrin-mediated endocytosis [22]. Remarkably, endosomal trafficking of LCMV and LASV was only mildly affected by dominant negative (DN) mutants of the small GTPase Rab5, involved in vesicular trafficking from the plasma membrane to the early endosome, and DN Rab7, which is implicated in delivery from early to late endosomes [19], [21]. Here we sought to identify cellular factors involved in cell entry and endosomal trafficking of LASV and LCMV.

## Results

### Cell entry of LASV and LCMV depend on microtubular transport

The low fusion pH of LASV and LCMV [18] suggests that the fusion event occurs in a late endosomal/lysosomal compartment. Since late endosomal compartments are located in the perinuclear area of the cell distant from the plasma membrane, delivery of vesicles to late endosomes is frequently facilitated by the cell's microtubular transport systems [12]. Depolymerization of microtubules by nocodazole results in accumulation of endocytosed cargo beyond early endosomes, but before late endosomes. Previous experiments demonstrated that nocodazole treatment markedly reduced infection of cells with LCMV and recombinant LCMV expressing the glycoprotein of LASV (rLCMV-LASVGP) [21]. These studies reported an inhibitory effect of nocodazole on early virus infection when the drug was added up to 60 minutes after infection [21]. Since Old World arenaviruses escape from endosomes already after circa 20 minutes [19], [21], these studies could not distinguish between effects of nocodazole on viral entry and/or early replication. To specifically address the role of microtubules in cell entry of LASV and LCMV prior to pH-dependent membrane fusion, we exploited the fact that cytosolic transport and replication of the nucleocapsid of vesicular stomatitis virus (VSV) after fusion does not require microtubules [23]. Since host cell attachment and entry of arenaviruses are mediated exclusively by the viral GP, recombinant VSV pseudotyped with arenavirus GPs allow separating arenavirus entry from subsequent steps of replication of the VSV core.

Recombinant VSV, whose G protein has been replaced by a GFP reporter (rVSVΔG) [24] was pseudotyped with the GPs of LASV and the LCMV isolate clone-13, henceforth referred to as LCMV, as described [25]. The virion particles of VSV and arenaviruses differ significantly in size and shape. Old World arenaviruses are pleomorphic particles ranging in size from 40–200 nm [11], whereas the rhabdovirus VSV has a characteristic bullet shape with a length of circa 180 nm and a width of 80 nm [26]. Previous studies showed that pseudotyping of VSV with arenavirus GPs preserved the bullet-shape of the VSV core [27]. Since the shape of virions may significantly affect the pathway of cell entry, we wanted to ensure that cell entry of the VSV pseudotypes rVSVΔG-LASVGP and rVSVΔG-LCMVGP still followed the unusual route of endocytosis described for LCMV and LASV. For comparison, we used a VSV pseudotype bearing the endogenous VSV G (rVSVΔG-VSVG) that enters cells by clathrin-mediated endocytosis (CME) [28] analogous to wild-type VSV [29].

**Figure 1:**
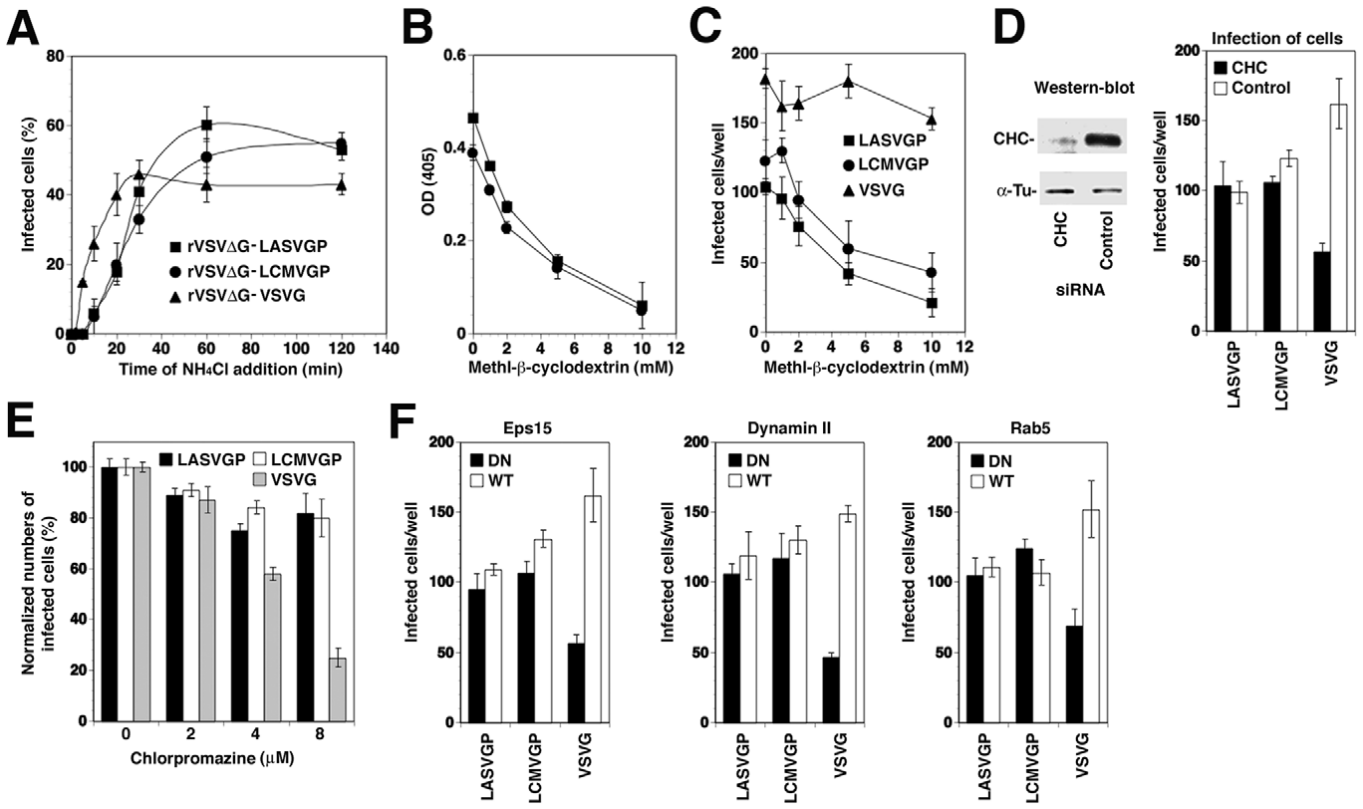
Recombinant VSV pseudotypes adopt the cell entry characteristics of LASV and LCMV. (A) Entry kinetics of rVSVΔG-LASVGP, rVSVΔG-LCMVGP, and rVSVΔG-VSVG. A549 cells were seeded in 96 well plates (10^4^ cells/well) and cultured for 16 hours. The resulting cell monolayers were chilled on ice and the indicated pseudotypes added at MOI of 3 for 1 hour. Unbound virus was removed and cells quickly shifted to 37°C. At the indicated time points 20 mM ammonium chloride (NH_4_Cl) was added and left throughout the experiment. After a total of 12 hours, cells were detached, fixed, and infection detected by the GFP reporter in flow cytometry. Data presented show percent GFP positive cells. Means (n = 3±SD). (B) Depletion of membrane cholesterol by MβCD. Monolayers of A549 cells were incubated with the indicated concentrations of MβCD for one hour and total cholesterol determined in a colorimetric assay. Shown are two independent experiments in triplicates expressed in percent of untreated control (mean ± SD). (C) Cholesterol depletion reduces infection with rVSVΔG-LASVGP, rVSVΔG-LCMVGP, but not rVSVΔG-VSVG. A549 cells (10^4^ cells/well) were treated with the indicated concentrations of MβCD as in (B), followed by infection with 200 PFU of rVSVΔG-LASVGP (LASVGP), rVSVΔG-LCMVGP (LCMVGP), and rVSVΔG-VSVG (VSVG) per well. Infection was assessed after 12 hours by detection of GFP expressing cells in fluorescence microscopy (mean ± SD; n = 3). (D) Infection with rVSVΔG-LASVGP and rVSVΔG-LCMVGP does not depend on clathrin. A549 cells were transfected with either a pool of siRNA targeting clathrin heavy chain (CHC) (50 nM per siRNA; 200 nM total concentration) or control siRNA (200 nM). After 48 hours, cells were lysed and expression of CHC assessed by Western-blot. For normalization, α-tubulin (α-Tu) was detected. Cells transfected with siRNA to either CHC or control siRNAs (Control) for 48 hours were infected with 200 PFU of rVSVΔG-LASVGP (LASVGP), rVSVΔG-LCMVGP (LCMVGP), and rVSVΔG-VSVG (VSVG) per well. After 12 hours, cells were fixed and infection assessed by detection of GFP as in (C) (mean ± SD; n = 3). (E) Infection with arenavirus pseudotypes is not sensitive to chlorpromazine (CPZ). A549 cells were pre-treated with the indicated concentrations of CPZ for 2 hours, followed by infection with rVSVΔG-LASVGP (LASVGP), rVSVΔG-LCMVGP (LCMVGP), and rVSVΔG-VSVG (VSVG) at MOI of 3. After 12 hours, infection was assessed as in (A) (mean ± SD; n = 3). (F) Comparison of the effects of DN mutants of Eps15, dynamin II, and Rab5 on infection with VSV pseudotypes. HEK293 cells were transfected with GFP-tagged DN and wild-type (WT) Eps15, dynamin II, and Rab5 using nucleofection, resulting in >90% transfection efficiency, as assessed by detection of GFP. After 24 hours, cells were infected with rVSVΔG-LASVGP (LASVGP), rVSVΔG-LCMVGP (LCMVGP), and rVSVΔG-VSVG (VSVG) at 200 PFU/well. Infected cells were detected by IFA using a mAb to VSV M protein and a rhodamine-red-X conjugated secondary antibody. Specimens were examined using a fluorescence microscope with a narrow band-pass filter and VSV M positive cells scored (mean ± SD; n = 3).

First, we compared the entry kinetics of rVSVΔG-LASVGP, rVSVΔG-LCMVGP and rVSVΔG-VSVG. To assess how fast receptor-bound VSV pseudotypes trafficked to endosomes and underwent membrane fusion, we determined the time required for the viruses to become resistant to the lysosomotropic agent ammonium chloride. When added to cells at a concentration of 20 mM, ammonium chloride raises the endosomal pH rapidly and blocks low pH-dependent cellular processes without causing overall cytotoxicity [30], [31]. Viruses were added to A549 human lung epithelial cells in the cold to allow receptor binding without internalization. The temperature was rapidly shifted to 37°C and 20 mM ammonium chloride added at the indicated time points and left throughout the experiment. After 12 hours cells were fixed and infected cells expressing GFP quantified by flow cytometry. In line with previous reports on VSV cell entry [29], 50% of rVSVΔG-VSVG reached insensitivity to ammonium chloride after only 8–10 minutes (Fig. 1A). In contrast, rVSVΔG-LASVGP and rVSVΔG-LCMVGP became insensitive to ammonium chloride with a half-time of circa 20 minutes, consistent with the entry kinetics of rLCMV-LASVGP [21] and LCMV [19].

To verify the use of distinct pathways of cell entry by rVSVΔG-LASVGP and rVSVΔG-LCMVGP on the one hand and rVSVΔG-VSVG on the other hand, we used a combination of pharmacological inhibitors, RNA interference (RNAi) and dominant negative (DN) mutants of regulatory proteins of endocytosis. Cell entry of LASV and LCMV is sensitive to drugs that deplete membrane cholesterol, such as methyl-β-cyclodextrin (MβCD), whereas VSV infection via CME is less sensitive [20], [32], [33]. Pre-treatment with increasing concentrations of MβCD efficiently depleted membrane cholesterol (Fig. 1B) and resulted in a dose-dependent reduction of infection with rVSVΔG-LASVGP and rVSVΔG-LCMVGP, but not rVSVΔG-VSVG (Fig. 1C). Cholesterol-dependence of cell entry is an operational definition and does not imply a specific pathway of entry. To address the specific role of CME in cell entry of the VSV pseudotypes, we depleted clathrin heavy chain (CHC) in A549 cells by RNAi as described [21], which resulted in >90% reduction of CHC protein levels after 48 hours as assessed by Western-blot (Fig. 1D). Subsequent infection of CHC-depleted cells revealed a significant reduction of infection with rVSVΔG-VSVG, but not rVSVΔG-LASVGP and rVSVΔG-LCMVGP, consistent with the clathrin-independent cell entry of LASV and LCMV (Fig. 1D). As a complementary approach, we performed inhibition studies using chlorpromazine (CPZ), a drug that perturbs the assembly of clathrin-coated pits at the plasma membrane and inhibits CME. Treatment of A549 cells with up to 8 µM CPZ did not significantly affect infection with rVSVΔG-LASVGP and rVSVΔG-LCMVGP, but lead to a dose-dependent reduction of rVSVΔG-VSVG infection (Fig. 1E). In addition we employed well-characterized dominant-negative (DN) mutants for regulatory proteins associated with CME, including a DN form of the clathrin-coat associated protein Eps15 (Eps15Δ95/295), which selectively perturbs CME without affecting clathrin-independent pathways [34], [35], and the DN mutant K44A of dynamin II [36], [37], [38]. The effects of the DN mutants on pseudotype infection were assessed as reported [21]. Briefly, HEK293 cells were transfected with GFP-tagged versions of the wild-type or DN mutants by nucleofection resulting in >90% transfected cells. Twenty hours post-transfection, cells were infected with VSV pseudotypes. At 12 hours p.i., cells were fixed and infected cells detected by immunofluorescence assay (IFA) using an antibody to the VSV M protein. As shown in Fig. 1F, expression of DN Eps15 and dynamin II markedly reduced infection with rVSVΔG-VSVG, but not the arenavirus pseudotypes. Another hallmark of LASV and LCMV cell entry is independence from Rab5 [19], [21], which is implicated in incoming vesicular trafficking from the plasma membrane to the early endosome [39], [40]. HEK293 cells were transfected with GFP-tagged constructs expressing wild-type and DN Rab5. Twenty hours later, cells were infected with VSV pseudotypes and infection assessed after 12 hours. In line with previous studies, infection with rVSVΔG-LASVGP and rVSVΔG-LCMVGP was only slightly affected by DN Rab5, whereas infection with rVSVΔG-VSVG was significantly reduced. In sum, the data confirm that rVSVΔG-LASVGP and rVSVΔG-LCMVGP enter cells via a cholesterol-dependent, clathrin-, and dynamin- independent pathway characteristic for cell entry of LASV and LCMV and bypasses Rab5-dependent transport to early endosomes. This makes our VSV pseudotypes suitable to address the specific role for microtubules and other cellular factors in LASV and LCMV cell entry.

**Figure 2:**
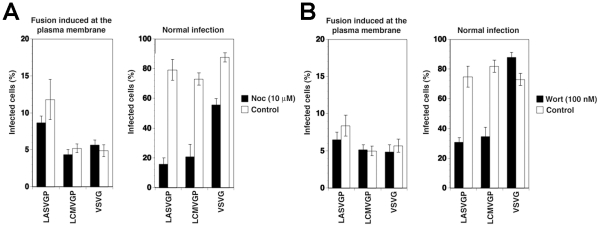
The role of microtubules and PI3K in cell entry of LASV and LCMV. (A) LASV and LCMV cell entry depend on microtubules. A549 cells (10^4^ cells/well) in 96 well plates were pre-treated with 10 µM nocodazole (Noc) for 1 hour, chilled on ice and incubated with rVSVΔG-LASVGP (LASVGP), rVSVΔG-LCMVGP (LCMVGP), and rVSVΔG-VSVG (VSVG) at MOI of 1 for one hour in the cold. Unbound virus was removed and cells shifted to 37°C in presence of nocodazole. To induce fusion at the plasma membrane, cells were pulsed with acidified medium (pH 5.0) for 15 minutes, followed by immediate neutralization. Cells subjected to normal infection were cultured in parallel, without applying the low pH pulse. After a total of 12 hours, infection assessed by detection of GFP expressing cells in flow cytometry as in Fig. 1A (mean ± SD; n = 3). (B) A role of PI3K in cell entry of LASV and LCMV. A549 cells were incubated with rVSVΔG-LASVGP (LASVGP), rVSVΔG-LCMVGP (LCMVGP), and rVSVΔG-VSVG (VSVG) at MOI of 1 for one hour in the cold and unbound virus removed. Cells were shifted to 37°C and fusion at the membrane was induced with a low pH pulse with acidified medium (pH 5.0) for 15 minutes. After neutralization, cells were cultured for a total of 12 hours in presence of 100 nM wortmannin. Cells subjected to normal infection were pre-incubated with virus in the cold. After removal of unbound virus, cells were rapidly shifted to 37°C for 5 minutes to allow internalization of the virus. Wortmannin was then added (100 nM) and left throughout the experiment. Infection was determined by detection of GFP expressing cells by flow cytometry as in Fig. 1A (mean ± SD; n = 3).

To verify independence of cytosolic transport and replication of the VSV nucleocapsid from microtubules, we artificially induced fusion directly at the plasma membrane. For this purpose, the VSV pseudotypes were attached to cells in the cold, unbound virus removed, and cells rapidly shifted to 37°C, immediately followed by exposure to acidified medium (pH = 5.0). After 15 minutes, cells were washed with neutral medium and virus infection determined 12 hours later by detection of the GFP reporter in flow cytometry. Consistent with published data [23], fusion of VSV pseudotypes at the plasma membrane resulted in relatively inefficient infection (<10% of normal infection levels) that was insensitive to nocodazole (Fig. 2A). When allowed to enter via their normal routes, infection of all pseudotypes was significantly affected by nocodazole (Fig. 2A), although to a different extent. Infection with rVSVΔG-LASVGP and rVSVΔG-LCMVGP was reduced by circa 80%, whereas infection with rVSVΔG-VSVG was less affected. The effect of nocodazole on infection with rVSVΔG-LASVGP and rVSVΔG-LCMVGP suggests that infection via the normal route involves microtubular transport to late endosomes where pH-dependent fusion occurs. The observed dependence of rVSVΔG-VSVG on microtubules is in line with previous reports that demonstrated a requirement for late endosomes transport for optimal VSV infection [23]. The significantly stronger effect of nocodazole on the arenavirus pseudotypes when compared to rVSVΔG-VSVG (Fig. 2A) may be explained by the fact that the G of VSV has a fusion pH of >6.0, allowing at least partial fusion and exit of the virus at the level of the early endosome, whereas LASVGP and LCMVGP cannot undergo fusion before reaching the more acidic late endosome.

### LASV and LCMV entry depends on phosphatidylinositol 3-phosphate (PI3P) and lysobisphosphatidic acid (LBPA)

In the host cell, receptor proteins at the plasma membrane can be internalized and delivered to late endosomes/lysosomes via specialized endosomal compartments called multivesicular bodies (MVB) [41]. In the MVB, receptor proteins destined for transport to late endosomes are sorted into intraluminal vesicles (ILV) that give the organelle its particular multivesicular morphology. Sorting into ILV is mediated by a large supramolecular complex, the endosomal sorting complex required for transport (ESCRT) [41]. In a first attempt to address a possible role of the MVB in LASV and LCMV cell entry, we interfered with the synthesis of the lipid phosphatidylinositol 3-phosphate (PI3P), which is required for formation of functional MVBs [41]. Perturbation of MVB formation by treatment of cells with inhibitors of PI3 kinase (PI3K) does not affect infection with VSV [23], because VSV can undergo fusion at the early endosome and subsequent cytosolic transport and replication is independent of PI3K. To assess a possible effect of PI3K inhibition on LASV and LCMV cell entry we therefore employed again our VSV pseudotypes. As expected, addition of the PI3K inhibitor wortmannin (100 nM) had no significant effect of VSV pseudotype infection when fusion was induced at the plasma membrane (Fig. 2B). To study the effect of wortmannin on infection via the normal entry route, VSV pseudotypes were attached to A549 cells in the cold followed by a temperature shift to 37°C to allow internalization. To address the effect of wortmannin selectively on endosomal transport, but not internalization of the virus particles, the fast-acting drug was added 5 minutes after the temperature shift, a time point when most of the virus is already present in vesicles [17], [19]. While wortmannin slightly enhanced infection with rVSVΔG-VSVG, consistent with published data [23], infection with the arenavirus pseudotypes was significantly reduced (Fig. 2B), indicating that PI3K activity is required for efficient cell entry of LASV and LCMV pseudotypes.

**Figure 3:**
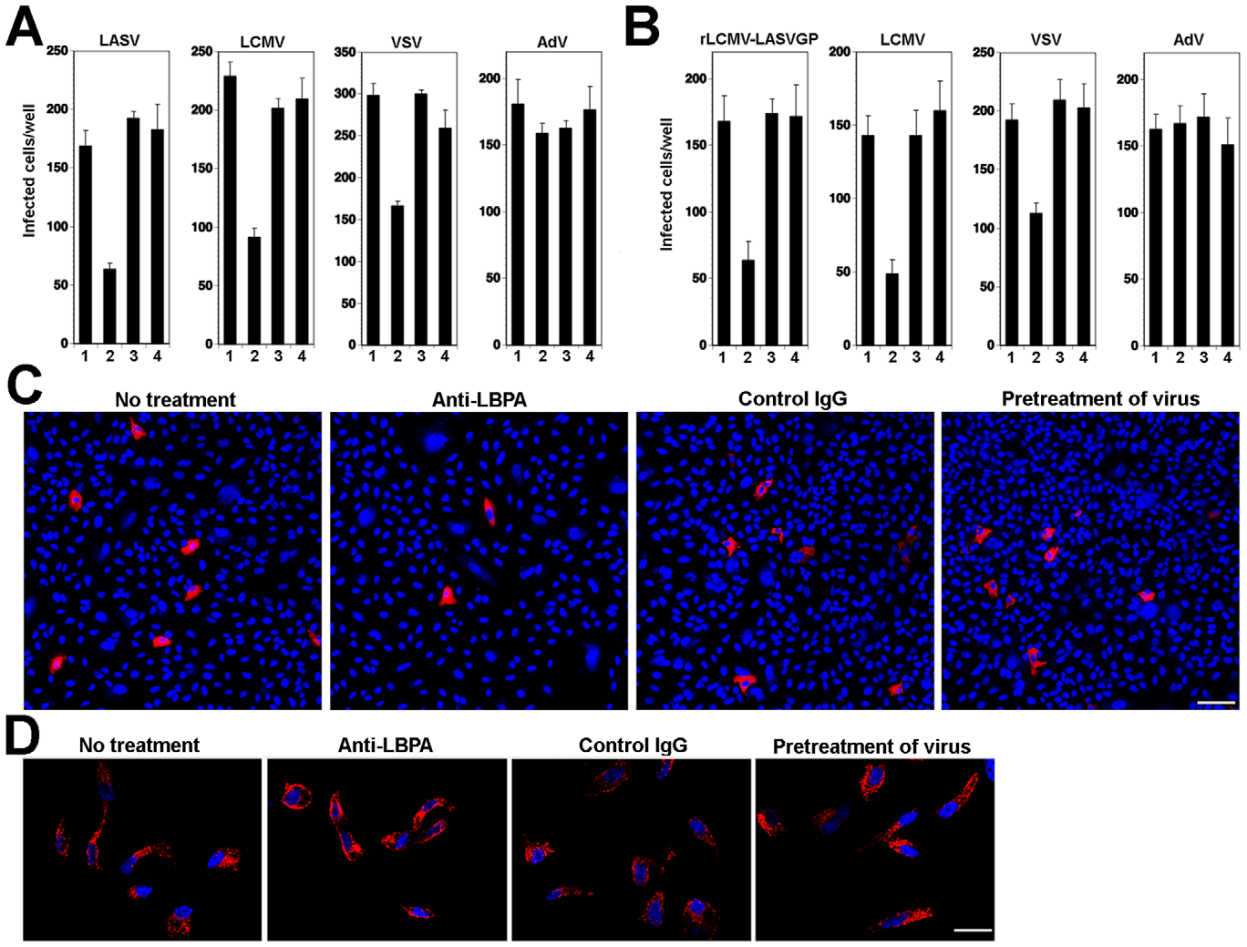
Cell entry of LASV and LCMV depends on LBPA. (A) Infection of VSV pseudotypes of LASV, LCMV, and VSV is affected by anti-LBPA antibody. A549 cells (10^4^ cells/well) in 96 well plates were pre-incubated with no antibody (1), 50 µg/ml mAb anti-LBPA (2) or isotype antibody control (3) for 14 hours. Cells were then infected with rVSVΔG-LASVGP (LASV), rVSVΔG-LCMVGP (LCMV), and rVSVΔG-VSVG (VSV), and AdV5-EGFP at 500 PFU/well. In specimens subjected to pretreatment only (4), the cells were incubated for 1 h at 4°C with viruses in presence of the Ab, unbound virus washed out, and cell incubated at 37°C in normal medium. Cells were fixed after 16 hours and EGFP positive cells counted (mean ± SD; n = 3). (B) Anti-LBPA perturbs infection of rLCMV-LASVGP and LCMV. Monolayers of A549 cells were treated as in (A), followed by infection with rLCMV-LASVGP, LCMV, and AdV5-EGFP (AdV) at 500 PFU/well. Cells were fixed after 16 hours (rLCMV-LASVGP, LCMV, VSV, or AdV5-EGFP) and 6 hours (VSV). Infection was detected with a mAb to LCMV NP (rLCMV-LASVGP, LCMV), VSV M protein (VSV), or EGFP (AdV5-EGFP) (mean ± SD; n = 3). (C) Representative specimens of cells infected with rLCMV-LASVGP in (B) LCMV NP is in red and nuclei (DAPI) appear in blue. Bar = 50 µm. (D) Anti-LBPA treatment does not interfere with transferrin uptake. Cells treated as in (A) were incubated for 10 min at 37°C with serum-free medium containing 20 µg/ml of Alexa594-labeled human transferrin, washed with ice cold PBS, acid-stripped to remove surface-bound transferrin, fixed, and the cellular distribution of transferrin (red) examined. Cell nuclei are stained in blue with DAPI (bar = 10 µM).

Another, more specific component involved in protein and lipid sorting through the MVB is the phospholipid lysobisphosphatidic acid (LBPA) that is found in late endosomes, in particular in the membrane of ILV, and is crucial for the ILV formation [42]. To address the role of LBPA in LASV and LCMV cell entry, we used the function-blocking monoclonal antibody (mAb) 6C4 to LBPA [42]. Pre-treatment of cells with anti-LPBA antibody allows uptake by fluid-phase endocytosis with accumulation in late endosomes [23]. A549 cells were treated with anti-LBPA antibody or an isotype (IgG1) control and subsequently infected with VSV pseudotypes of LASV and LCMV. As a positive control, we used VSV pseudotypes bearing VSVG, whose infection is perturbed by anti-LBPA treatment [23]. For comparison, we included a replication-deficient recombinant human species C adenovirus serotype 5 expressing EGFP (AdV5-EGFP) [43], which naturally infects human lung epithelial cells, such as A549 cells [44]. In contrast to Old World arenaviruses, human species C adenoviruses (AdV2, AdV5) use the Coxsackie virus B adenovirus receptor (CAR) and integrins for host cell attachment and are internalized via a clathrin-dependent pathway [45]. Upon internalization, AdV2 and AdV5 are delivered to early endosomes and rapidly penetrate into the cytoplasm by a complex mechanism including low pH, interaction of the AdV penton base protein with αvβ5 integrins, integrin signaling, and activity of a viral protease [45]. To exclude a direct anti-viral effect of the antibody *per se*, untreated cells were incubated with the viruses for 1 h at 4°C in presence of the antibody, unbound virus washed out, and cell incubated at 37°C in absence of the antibody. As shown in Fig. 3A, pre-treatment of cells with anti-LBPA specifically reduced subsequent infection with VSV pseudotypes of LASV, LCMV, and VSV, but not AdV5-EGFP. Treatment of the virus with anti-LBPA antibody had no effect on subsequent infection.

Next, we sought to verify our findings with live arenaviruses. To circumvent biosafety restrictions associated with LASV, we studied LASV entry using a recombinant LCMV expressing LASV GP (rLCMV-LASVGP). Since LASV cell entry is mediated exclusively by the viral GP, rLCMV-LASVGP can be used as a BSL2 surrogate to study LASV cell entry in the context of a productive arenavirus infection [21]. A549 cells were pre-treated with anti-LBPA antibody as described above, followed by infection with rLCMV-LASVGP, LCMV, VSV, and AdV5-EGFP. To prevent secondary infection by virus released from infected cells, we added 20 mM ammonium chloride at 4 hours after infection. Cells infected with rLCMV-LASVGP, LCMV, and AdV5-EGFP were fixed after 16 hours and cells infected with VSV after 6 hours. Infection with rLCMV-LASVGP and LCMV was detected by immunofluorescence assay (IFA) using antibodies to the LCMV nucleoprotein (NP) and VSV infection was detected by immunostaining with an antibody to the matrix (M) protein. Similar to our findings with the VSV pseudotypes, treatment with anti-LBPA antibody resulted in significant reduction of infection with rLCMV-LASVGP and LCMV, but not AdV5-EGFP (Fig. 3B). Remaining infected cells in cultures pre-treated with anti-LBPA showed normal morphology and expression levels of viral NP (Fig. 3C), excluding overt toxicity of antibody treatment. To further exclude a non-specific effect of anti-LBPA on endocytosis, we assessed the impact of anti-LBPA on the uptake of transferrin, which involves CME followed by delivery to the early endosome, but does not implicate the MVB/late endosome. Cells pre-treated with anti-LBPA showed normal internalization of transferrin (Fig. 3D) excluding general perturbation of endocytosis and membrane dynamics by the anti-LBPA antibody. In sum, the dependence of rLCMV-LASVGP and LCMV cell entry and productive infection on PI3K and LBPA provided the first hints that, upon internalization, the virus-receptor complex may undergo sorting in the MVB/late endosome.

### Cell entry of LASV and LCMV involves the ESCRT components Hrs, Tsg101, Vps22, and Vps24

**Figure 4:**
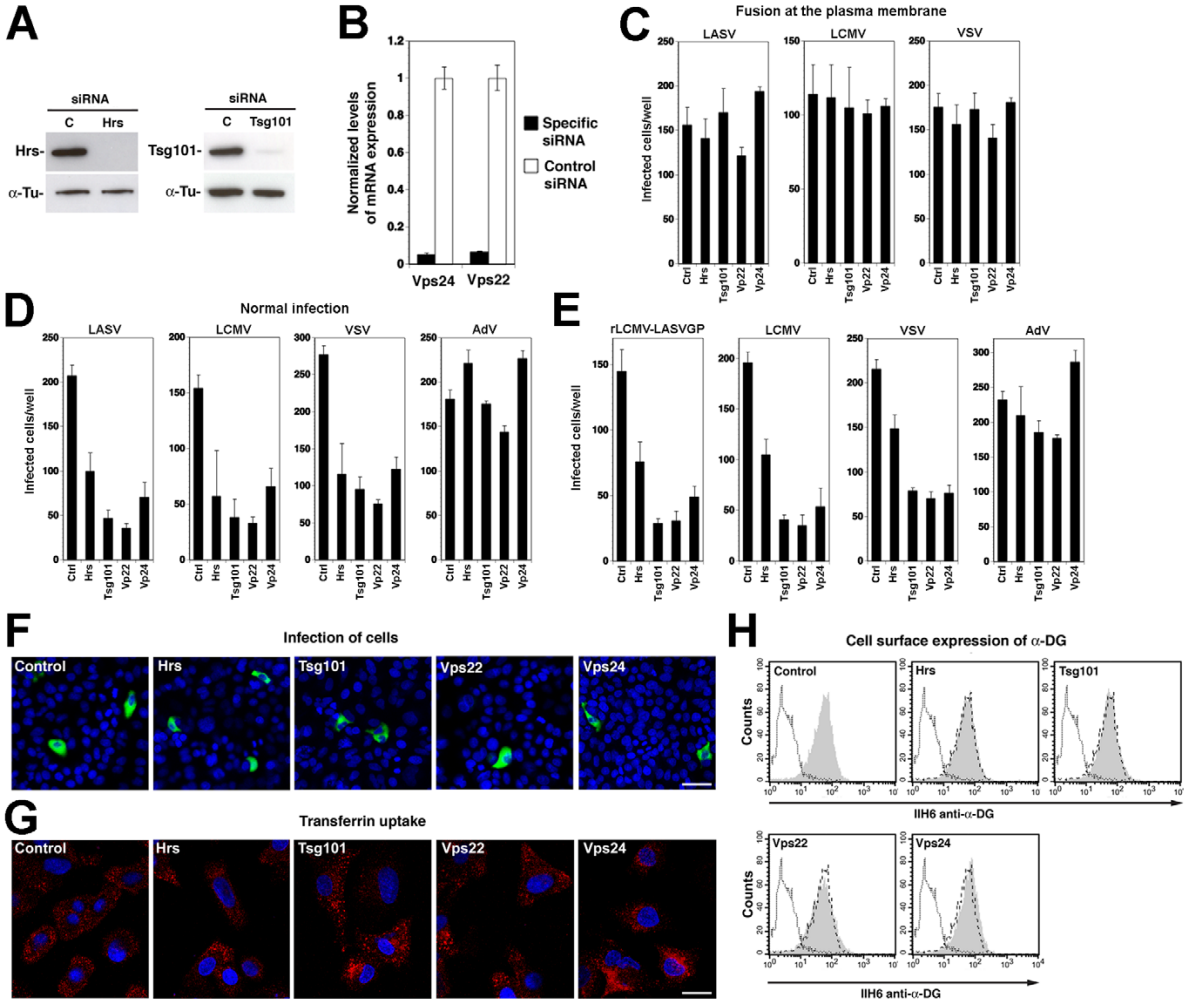
A role for Hrs, Tsg101, Vps22, and Vps24 in cell entry of LASV and LCMV. (A) A549 cells were transfected with siRNAs specific for Hrs and Tsg101 or control siRNA and efficiency of depletion assessed after 72 hours by Western-blot. For normalization, α-tubulin (α-Tu) was used. (B) Cells were transfected with siRNAs for Vps22 and Vps24 or control siRNA and efficiency of depletion assessed after 72 hours by quantification of mRNA levels by RT-qPCR (for details see Methods). Given are normalized levels of mRNA expression. (C) Depletion of Hrs, Tsg101, Vps22, and Vps24 does not affect post-entry steps of VSV pseudotype infection. A549 cells were treated with specific siRNAs to Hrs, Tsg101, Vps22, and Vps24 and control siRNA for 48 hours. Cells were then detached and reseeded in 96 well plates (10^4^ cells/well). After 24 hours, cells were incubated with rVSVΔG-LASVGP (LASV) (4000 PFU/well), rVSVΔG-LCMVGP (LCMV) (4000 PFU/well), and rVSVΔG-VSVG (VSV) (2000 PFU/well) in the cold, followed by washing and a low pH pulse to induce fusion at the plasma membrane as in Fig. 2. Upon neutralization, cells were fixed after 16 hours and EGFP positive cells scored (mean ± SD; n = 3). (D, E) Depletion of Hrs, Tsg101, Vps22, and Vps24 affects cell entry of LASV and LCMV. Cells subjected to RNAi silencing as in (A) were infected via the normal route of entry (D) with rVSVΔG-LASVGP (LASV), rVSVΔG-LCMVGP (LCMV), rVSVΔG-VSVG (VSV), and AdV5-EGFP (AdV) (500 PFU/well) and (E) with rLCMV-LASVGP, LCMV, rVSVΔG-VSVG (VSV), and AdV5-EGFP (AdV) at 500 PFU/well. After 16 hours, cells were fixed and infection quantified by detection of EGFP positive cells (D) or LCMV NP and EGFP (E) as in Fig. 3 (mean ± SD; n = 3). (F) Representative specimens of rLCMV-LASVGP infected cells in cultures treated with the indicated siRNAs in (E). LCMV NP is stained in green and cell nuclei stained with DAPI appear in blue (bar = 20 µM). (G) Depletion of Hrs, Tsg101, Vps22, and Vps24 by RNAi does not interfere with transferrin uptake. Cells were treated as in (A, B) and transferrin uptake assessed as in Fig. 3D. Transferrin is in red and cell nuclei in blue (bar = 10 µM). (H) Depletion of Hrs, Tsg101, Vps22, and Vps24 by RNAi does not affect cell surface expression of α-DG. Cells were subjected to RNAi as in (A, B). After 72 hours cell surface staining was performed with mAb IIH6 to α-DG, combined with Alexa 594 secondary antibody. Data were acquired in a FACSCalibur flow cytometer and analyzed using Cell Quest software. In histograms, y-axis represents cell numbers and x-axis Alexa 594 fluorescence intensity. Shaded area: primary and secondary antibody, empty area: secondary antibody only. The broken line indicates the superimposition of the shaded peak (primary+secondary antibody) from cells treated with control siRNA. Note that treatment with siRNAs to Hrs, Tsg101, Vps22, and Vps24 does not significantly alter the mean fluorescence intensity of the IIH6 signal.

Sorting of cargo into ILV membranes of the MVB involves the initial recognition of cargo by the ESCRT-0 protein Hrs, which results in sequential assembly of ESCRT-I, ESCRT-II, and ESCRT-III complexes, followed by vesicle formation [41]. In a first step to investigate the role of the ESCRT for LASV and LCMV entry, we performed RNAi silencing for selected components of the different ESCRT sub-complexes: the ESCRT-0 component Hrs, the pivotal ESCRT-I protein Tsg101, and the proteins Vps22 and Vps24 that are crucial components of ESCRT-II and ESCRT-III, respectively. Hrs regulates MVB formation by recruiting the ESCRT to endosomes [46] and Tsg101 is a crucial component of the ESCRT involved in sorting of cargo into ILV, as well as ILV formation [47], [48]. Vps22 and Vps24 have been implicated in endosomal sorting and degradation of cellular membrane receptors [49], [50]. For RNAi silencing, firmly established and validated siRNA sequences to human Hrs, Tsg101, Vps22, and Vps24 were used (for details see Methods). Silencing of Hrs and Tsg101 in A549 cells by specific siRNAs resulted in efficient depletion of the endogenous proteins as assessed by Western-blot (Fig. 4A). Due to the lack of specific antibodies to Vps22 and Vps24, the efficiency of RNAi was verified by quantitative PCR that revealed >90% reduction in mRNA levels after 72 hours (Fig. 4B).

To assess their role in LASV and LCMV cell entry, A549 cells were treated with siRNAs to Hrs, Tsg101, Vps22, Vps24, and a control siRNA. After 72 hours, cells were infected with VSV pseudotypes of LASV and LCMV, VSV pseudotypes bearing VSVG, and AdV5-EGFP. To exclude possible effects of the siRNA treatment on the cytoplasmic transport and/or replication of the VSV core, we included a series in which fusion of VSV pseudotypes was induced at the plasma membrane as described above. Upon fusion at the plasma membrane, infection of the VSV pseudotypes was unaffected by siRNA treatment (Fig. 4C), excluding adverse effects of RNAi on VSV replication at a post-entry step. Next, we infected siRNA treated cells via the normal route of cell entry. Although Tsg101 has previously been implicated in budding of arenaviruses [51], this was not a concern in our experimental setup, as VSV pseudotypes are replication-deficient, preventing secondary infection. After 16 hours of infection, cells were fixed and infection assessed by detection of the GFP reporter. When using the normal route of cell entry, depletion of Hrs, Tsg101, Vps22, and Vps24 resulted in significant reduction in numbers of infected cells with VSV pseudotypes of LASV, LCMV, and VSV (Fig. 4D). In contrast, infection with AdV5-EGFP was only mildly affected by depletion of Tsg101 and Vps22, whereas silencing of Hrs and Vps24 enhanced infection (Fig. 4D). Since depletion of Hrs, Tsg101, Vps22, and Vps24 did not affect post entry-steps of VSV infection (Fig. 4C) the data suggest a role for these ESCRT components in cell entry of pseudotypes of LASV and LCMV. To validate our results with arenaviruses, A549 cells were subjected to siRNA treatment, followed by infection with rLCMV-LASVGP and LCMV, using VSV pseudotypes bearing VSVG and AdV5-EGFP as positive and negative controls, respectively. Since Tsg101 has previously been implicated in budding of arenaviruses [51], we addressed the effect of the knockdowns on viral entry by blocking secondary infection with 20 mM ammonium chloride added at 4 hours post infection. After 16 hours of infection, cells were fixed and infected cells detected by IFA using antibodies to LCMV NP. Consistent with the results obtained with VSV pseudotypes (Fig. 4D), depletion of Hrs, Tsg101, Vps22, and Vps24 resulted in significant reduction of the numbers of infected cells with all viruses, except AdV5-EGFP (Fig. 4E). In cultures treated with siRNAs specific to Hrs, Tsg101, Vps22, and Vps24 residual infected cells had similar expression levels of LCMV NP as observed in cells treated with control siRNA (Fig. 4F), indicating that the siRNA treatment did not affect post-fusion steps of early replication. To exclude unspecific off-target effects on membrane dynamics and endocytosis, cells subjected to RNAi for Hrs, Tsg101, Vps22, and Vps24 were tested for transferrin uptake. None of the RNAi treatments significantly affected the ability of cells to internalize transferrin, excluding a general impact on endocytosis (Fig. 4G). Since perturbation of the ESCRT machinery may affect cellular trafficking of the Old World arenavirus receptor, we verified the cell surface expression of α-DG. Cells subjected to RNAi were examined by cell surface staining with mAb IIH6 that specifically recognizes the mature, functionally glycosylated form of the receptor [52]. As shown in Fig. 4H, depletion of Hrs, Tsg101, Vps22, and Vps24 did not affect the cell surface levels of functional α-DG in A549 cells.

**Figure 5:**
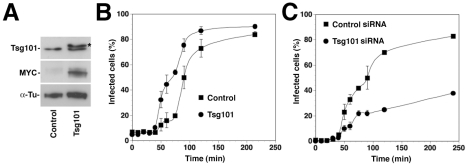
Overexpression of Tsg101 accelerates entry kinetics of rLCMV-LASVGP. (A) Over-expression of a MYC-tagged recombinant Tsg101 by transient transfection of HEK293 cells. Cells were transfected with recombinant Tsg101 (Tsg101) or empty vector (control). After 36 hours total cell lysates were probed by Western-blot with antibodies recognizing Tsg101 or the MYC epitope. The MYC-tagged form of Tsg101 runs at a slightly higher apparent molecular mass, due to the presence of the tag (*). (B) Overexpression of Tsg101 accelerates the entry kinetics of rLCMV-LASVGP. Cells transfected with recombinant Tsg101 (Tsg101) or empty vector (Control) as in (A) were cooled on ice for 30 min and rLCMV-LFVGP added at an MOI of 3. After incubation for 1 h on ice, unbound virus was washed off, and cells shifted to 37°C. At different time points, medium containing 20 mM ammonium chloride was added. After a total of 16 h, cells were fixed and LCMVNP detected by intracellular staining with mAb 113 to LCMV NP, combined with Alexa 488-labeled secondary antibody. NP positive cells were quantified by flow cytometry as in Fig. 5D. Note the delay in the fusion kinetics relative to Fig. 1A due to incubations of cells with ice cold medium after the washes. Data presented are means (n = 3+SD). (C) Entry kinetics in cells depleted of Tsg101. HEK293 cells were transfected with siRNA to Tsg101 or control siRNA as in Fig. 5A, resulting in >90% reduction of Tsg101 protein expression after 72 hours (data not shown). Cells treated with siRNA for 72 hours were cooled on ice for 30 minutes and rLCMV-LFVGP added at an MOI of 3. After removal of unbound virus entry kinetics was assessed as in (B). Data presented are means (n = 3+SD).

To confirm the role of Tsg101 on LASV and LCMV cell entry in a complementary manner, we overexpressed recombinant Tsg101 and assessed the impact on the infection kinetics by ammonium chloride treatment, as described above. For over-expression studies, a high transfection efficiency of >80% was needed. As A549 cells were difficult to transfect, with transfection efficiencies of <40%, we used HEK293 cells for these studies. Depletion of Hrs, Tsg101, Vps22, and Vps24 in HEK293 affected infection with rLCMV-LASVGP and LCMV in a similar manner as observed in A549 cells (data not shown). Briefly, HEK293 cells were transfected with an expression plasmid encoding a recombinant MYC-tagged form of Tsg101 or empty vector, resulting in significant over-expression of Tsg101 (Fig. 5A). After 36 hours, cells were incubated with rLCMV-LASVGP in the cold, unbound virus removed, and the temperature shifted to 37°C. At different time points 20 mM ammonium chloride was added and virus infection detected after a total of 16 hours by intracellular staining for LCMV NP and flow cytometry. Over-expression of Tsg101 significantly shortened the half time by which the virus infection became insensitive to ammonium chloride (Fig. 5B). However, the total number of infected cells was only slightly increased (Fig. 5B), indicating a selective acceleration of the viral entry process. Consistent with the data obtained in A549 cells (Fig. 4E), depletion of Tsg101 by RNAi markedly reduced the number of infected cells (Fig. 5C). The faster apparent kinetics of viral entry upon Tsg101 overexpression further supports a role of this ESCRT-I protein as a positive regulator for Old World arenavirus entry.

### rLCMV-LASVGP and LCMV infection involves the ESCRT-associated ATPase Vps4

**Figure 6:**
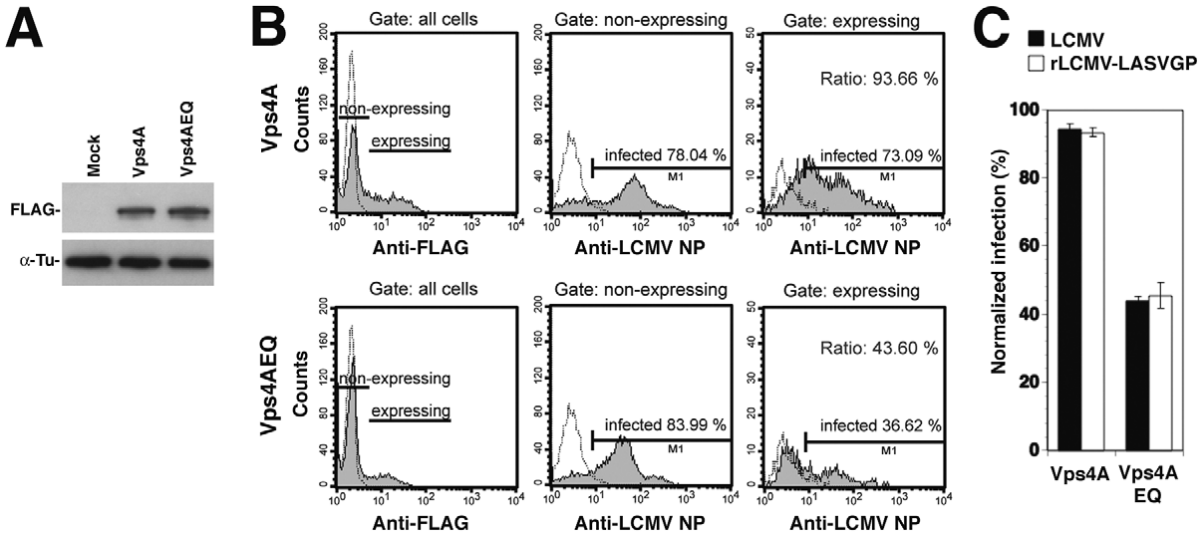
A role of Vps4 in rLCMV-LASVGP and LCMV entry. (A) HEK293 cells were transiently transfected with FLAG-tagged wild-type Vps4A and the DN mutant Vps4AQE and expression of the recombinant proteins detected in Western-blot. (B) Expression of DN Vps4A reduced viral infection. Cells were transiently transfected with FLAG-tagged wild-type and DN Vps4A. After 36 hours, cells were infected with rLCMV-LASVGP and LCMV (MOI = 1). After 16 hours, cells were fixed and stained for the Vps4A variants (anti-FLAG) and LCMV NP. Cells were analyzed by FACS separating transfected “expressing” from untransfected “non-expressing” cells with gating based on the intensity of the anti-FLAG signal. The percentage of cells infected within each population was quantified. (C) Quantitation of (B). All data presented are means (n = 3 ± SD).

To further investigate a possible role of ILV in rLCMV-LASVGP and LCMV cell entry, we targeted the ESCRT-associated ATPase Vps4 A and B, which are implicated in the final step of the fission of ILV in MVBs in mammalian cells [41]. To this end we employed an ATP hydrolysis DN mutant of Vps4A, Vps4AEQ, which interferes with the formation of functional Vps4 oligomers and perturbs the formation of ILV mediated by both Vps4A and B [53], [54], [55]. HEK293 cells were transfected with FLAG-tagged versions of wild-type Vps4A and the DN mutant, resulting in comparable levels of expression detected in Western-blot (Fig. 6A). Thirty-six hours post transfection cells were infected with rLCMV-LASVGP and LCMV. Since Vps4 has been identified as a cellular factor required for arenavirus budding [56], we again added 20 mM ammonium chloride at 4 hours p.i. to prevent secondary infection. At 16 hours post infection, cells were fixed and analyzed by FACS for viral antigen expression. Live cells expressing similar levels of the FLAG-tagged wild type and DN mutants were selected and those expressing viral antigen scored (Fig. 6B). Over-expression of DN Vps4A, but not the wild-type control resulted in a specific reduction of infection with rLCMV-LASVGP and LCMV (Fig. 6C). Together, our data provide the first evidence that cell entry of rLCMV-LASVGP and LCMV requires a functional MVB.

### Incoming LCMV transiently co-localizes with Tsg101 prior to delivery to late endosomes

**Figure 7:**
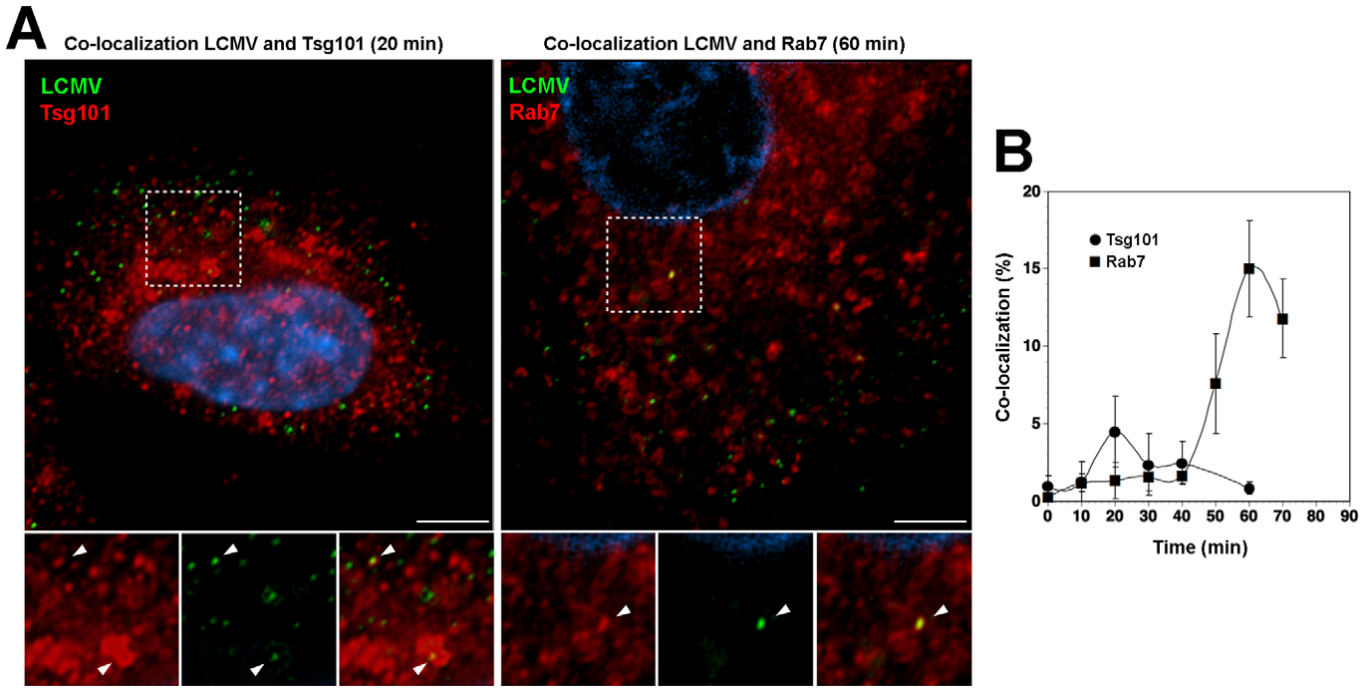
Incoming LCMV transiently co-localized with Tsg101 prior to reaching late endosomes. (A) Co-localization of LCMV WE54 with Tsg101 and Rab7. A549 cells were cooled on ice for 30 min and LCMV WE54 added at an MOI of ∼100. After incubation for 1 hour on ice, unbound virus was washed off, cells shifted to 37°C and fixed at the indicated time points. Cells were then immunostained to detect endogenous Tsg101 or Rab7 and incoming viral particles. Representative images are shown. Left: LCMV (green) and Tsg101 (red) at 20 min after temperature shift; right: LCMV (green) and Rab7 (red) at 60 min after temperature shift. Scale bar = 5 µm. (B) Quantification of co-localization. Ten randomly selected cells per time point were analyzed by confocal microscopy and the percentage of co-localizing viruses determined as described in Materials and Methods. Data presented are means ± SD (n = 10).

Using a combination of RNAi and recombinant proteins we provided first evidence for a role of the ESCRT in cell entry of the Old World arenaviruses LASV and LCMV. To corroborate these findings, we performed co-localization studies of the prototypic LCMV with Tsg101, which represents a key component of ESCRT-I implicated in recognition of cargo, as well as Rab7, used as a marker for late endosomes. For our co-localization experiments, purified virus had to be used at high MOI. Based on its better in vitro growth properties, we used the LCMV isolate WE54 for these studies. LCMV WE54 had been used for the initial characterization of the LCMV cell entry pathway [19] and shares the receptor binding characteristics and entry pathway of LCMV cl-13 and LASV [20]. LCMV WE54 was grown in large quantities in BHK21 cells and purified by ultracentrifugation on a renografin gradient as described [57]. To investigate whether incoming LCMV co-localized with Tsg101, A549 cells were exposed to purified LCMV WE54 (MOI = 100) at 4°C. Unbound virus was removed and temperature shifted to 37°C. Cells were fixed at different times and immunostaining performed. After mild permeabilization of cells, incoming virus was detected with an antibody to NP, labeling the virus RNP core. Endogenous Tsg101 was detected with a specific antibody. As a marker for late endosomal compartments, we detected endogenous Rab7. Although dominant negative Rab7 did not block cell entry of LCMV and LASV [19], [21], previous co-localization studies demonstrated accumulation of LCMV in Rab7 positive compartment after 20 minutes, suggesting virus escape from late endosomes [19]. Specimens were examined by confocal laser scanning microscopy and the co-localization of the virus with Tsg101 and Rab7 assessed as described in Materials and Methods. As shown in Fig. 7, we observed a transient co-localization of the virus with Tsg101 with a maximum at 20 minutes after the temperature shift. At this early time point co-localization of the virus with Rab7 was still weak (Fig. 7B). At later time points, the co-localization between virus and Tsg101 was gradually lost with a concomitant increase of co-localization with Rab7 (Fig. 7B). The relatively low extent of co-localization of LCMV with Tsg101 may be due to a transient interaction of Tsg101 with the virus. Together, our data indicate that incoming virus transiently passes through an MVB compartment associated with Tsg101, and is then rapidly delivered to late endosomes, consistent with sorting by the ESCRT.

### LASV and LCMV cell entry depends on the ESCRT-associated protein Alix

**Figure 8:**
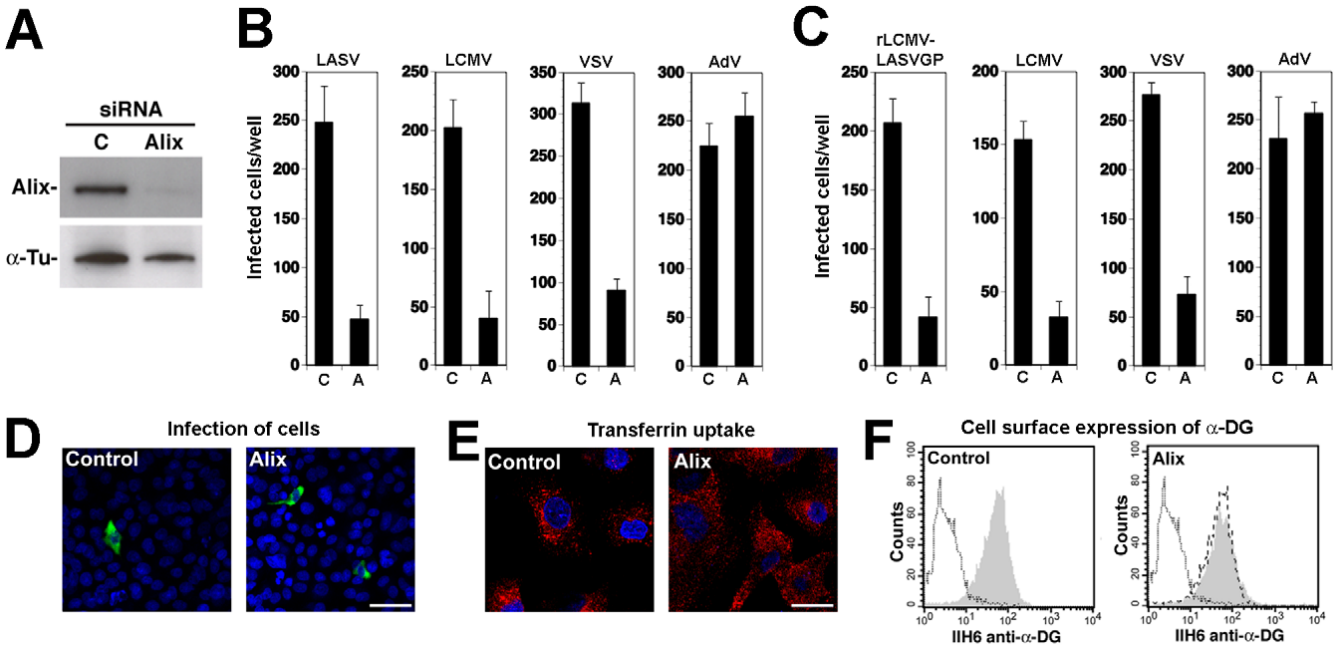
Alix is required for cell entry of LASV and LCMV. (A) A549 cells were transfected with siRNAs specific for Alix or control siRNA and efficiency of depletion assessed after 72 hours by Western-blot. For normalization, α-tubulin (α-Tu) was used. (B, C) Depletion of Alix perturbed cell entry of LASV and LCMV. A549 cells were treated with specific siRNAs to Alix and control siRNA as in (A), followed by infection with (B) rVSVΔG-LASVGP (LASV), rVSVΔG-LCMVGP (LCMV), rVSVΔG-VSVG (VSV), and AdV5-EGFP (AdV) at 500 PFU/well or (C) rLCMV-LASVGP, LCMV, rVSVΔG-VSVG (VSV), and AdV5-EGFP (AdV) (500 PFU/well). Cells were fixed after 16 hours and infection detected by IFA as in Fig. 4 (mean ± SD; n = 3). (D) Representative specimens of rLCMV-LASVGP infected cells in cultures treated with the indicated siRNAs in (C). LCMV NP is in green and cell nuclei in blue (bar = 20 µM). (E) Depletion of Alix does not interfere with transferrin uptake. Cells treated as in (A) were subjected to transferrin uptake assay as in Fig. 3D. The cellular distribution of transferrin (red) and cell nuclei stained with DAPI in blue are shown (bar = 10 µM). (F) Depletion of Alix does not affect cell surface expression of α-DG. Cells were subjected to RNAi as in (A) and cell surface staining for α-DG performed after 72 hours as in Fig. 4H. In histograms, y-axis represents cell numbers and x-axis Alexa 594 fluorescence intensity. Shaded area: primary and secondary antibody, empty area: secondary antibody only. The broken line indicates the superimposition of the shaded peak (primary+secondary antibody) from cells treated with control siRNA.

The apoptosis linked gene (ALG)-2-interacting protein X (Alix) interacts with ESCRT proteins [58], [59], is involved in endosomal membrane dynamics in a LBPA-dependent manner [23], [42], [60], membrane receptor endocytosis [61], [62], and viral budding [63], [64], [65]. To address the role of Alix in cell entry of LASV and LCMV, we performed RNAi silencing of the protein in A549 cells using a validated siRNA to human Alix. Depletion of Alix by the specific siRNA resulted in >90% reduction of Alix protein levels after 72 hours, as assessed by Western-blot (Fig. 8A). Cells depleted of Alix by RNAi were infected with VSV pseudotypes of LASV, LCMV, and VSV as well as AdV5-EGFP. As shown in Fig. 8B, depletion of Alix markedly reduced infection with all VSV pseudotypes, but not AdV5-EGFP. When fusion was allowed at the plasma membrane, depletion of Alix had no effect on post entry replication of the VSV core (data not shown). The results obtained with the VSV pseudotypes were validated with rLCMV-LASVGP and LCMV (Fig. 8C). As observed with the RNAi silencing of ESCRT proteins, depletion of Alix significantly reduced the number of infected cells (Fig. 8C), but did not reduce the expression levels of LCMV NP in remaining infected cells (Fig. 8D). The efficient depletion of Alix did not cause overt toxicity and had no significant effect on the uptake of transferrin (Fig. 8E). Cell surface expression of functional α-DG was unaltered (Fig. 8F). Similar effects of Alix depletion on infection with rLCMV-LASVGP, LCMV, and VSV were observed in the prototypic primate cell line CV-1 (data not shown), indicating a role of Alix in rLCMV-LASVGP and LCMV cell entry in other cell types.

### An LCMV isolate independent of α-DG also depends on ESCRT proteins for cell entry

**Figure 9:**
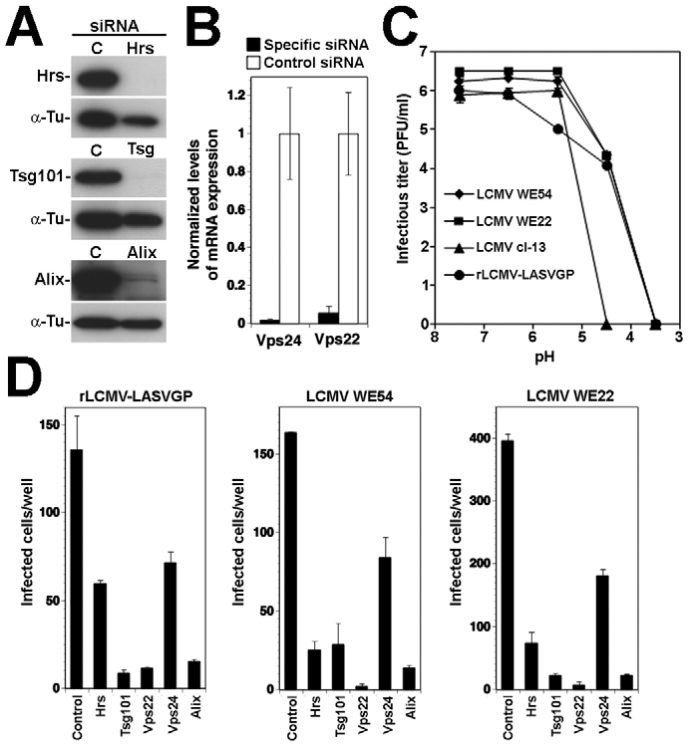
Infection of the α-DG independent LCMV WE22 also depends on ESCRT proteins. (A, B) Depletion of Hrs, Tsg101, Vps22, Vps24, and Alix. A549 cells were subjected to siRNA knock down of Hrs, Tsg101, Vps22, Vps24, and Alix as in Figs. 4A and 8A. Depletion of Hrs, Tsg101, and Alix was verified by Western blot as in 4A and 8A. The know-down of Vps22 and Vps24 was validated by detection of mRNA using RT-qPCR as in 4B. (C) LCMV WE22 retains the low fusion pH of WE54. LCMV WE54, WE22, cl-13 and rLCMV-LASVGP (10^7^ PFU/ml) were exposed to buffer solution with the indicated pH for 15 minutes. Samples were immediately neutralized and infectious virus titers determined by immunofocus assay on fresh monolayers of A549 cells. Means of 2 independent experiments are shown. (D) Infection with LCMV WE22 depends on Hrs, Tsg101, Vps22, Vps24, and Alix. A549 cells were subjected to RNAi silencing of Hrs, Tsg101, Vps22, Vps24, and Alix as in (A) and (B) respectively. After 72 hours cells were infected with the indicated viruses (500 PFU/well) and infection assessed after 16 hours by IFA (mean ± SD; n = 3).

While most strains and variants of LCMV use α-DG as a cellular receptor, there exist LCMV isolates that can use alternative receptors, different from α-DG [57], [66]. The LCMV isolates WE54 and WE22 differ from each other by only one point mutation in GP1, S153 in WE54 and F153 in WE22. However, the receptor use of WE54 and WE22 is strikingly different. Similar to LCMV cl-13 and LASV, LCMV WE54 binds α-DG with high affinity and depends on α-DG for cell entry, whereas WE 22 does not bind to α-DG and infects cells in an α-DG-independent manner [67]. The cellular receptor(s) of LCMV WE22 are currently unknown, but initial characterization revealed that they are either proteins or protein-associated structures [57]. Based on their close structural relationship and strikingly different receptor use, we utilized WE54 and WE22 to investigate the role of the ESCRT proteins and Alix in infection with an α-DG-independent LCMV variant. The mutation S153F that distinguished LCMV WE54 from WE22 lies within GP1, whereas membrane fusion of arenaviruses is mediated by the transmembrane GP2 moiety. However, we could at this point not exclude that the S153F mutation had an impact on the fusion pH of WE22, perhaps allowing the virus to exit at an earlier stage of the endosomal pathway. To assess the relative fusion pH of WE 54 and WE22, the viruses were exposed to progressively more acidic pH for 15 minutes. After neutralization, viruses were added to fresh monolayers of A549 cells and residual infections titers determined by immunofocus assay as described [68]. As controls, we used LCMV cl-13 and rLCMV-LASVGP. In line with published results [69], we observed a high resistance of rLCMV-LASVGP and LCMV cl-13 towards acidic pH, with significant inactivation of the virus only at pH<5.0 (Fig. 9A). LCMV WE54 and WE22 showed similar stability under low pH, indicating that the S153F mutation in GP1 did not significantly affect the fusion pH (Fig. 9A). Next, we tested the role of the ESCRT proteins Hrs, Tsg101, Vps22, Vps24, and the ESCRT-associated protein Alix in cell entry of LCMV WE22. A549 cells were subjected to RNAi silencing as described above, followed by infection with LCMV WE54, LCMV WE22, and rLCMV-LASVGP, which was used as a positive control. Similar to the results obtained with LCMV cl-13, infection of both LCMV WE54 and WE22 was significantly reduced in cells depleted of Hrs, Tsg101, Vps22, Vps24, and Alix (Fig. 9B), suggesting that α-DG dependent and α-DG independent LCMV isolates pass through the MVB and require the ESCRT machinery for cell entry.

### The role of the MVB/ESCRT for LASV and LCMV cell entry is conserved in human cells of the monocyte/macrophage lineage

**Figure 10:**
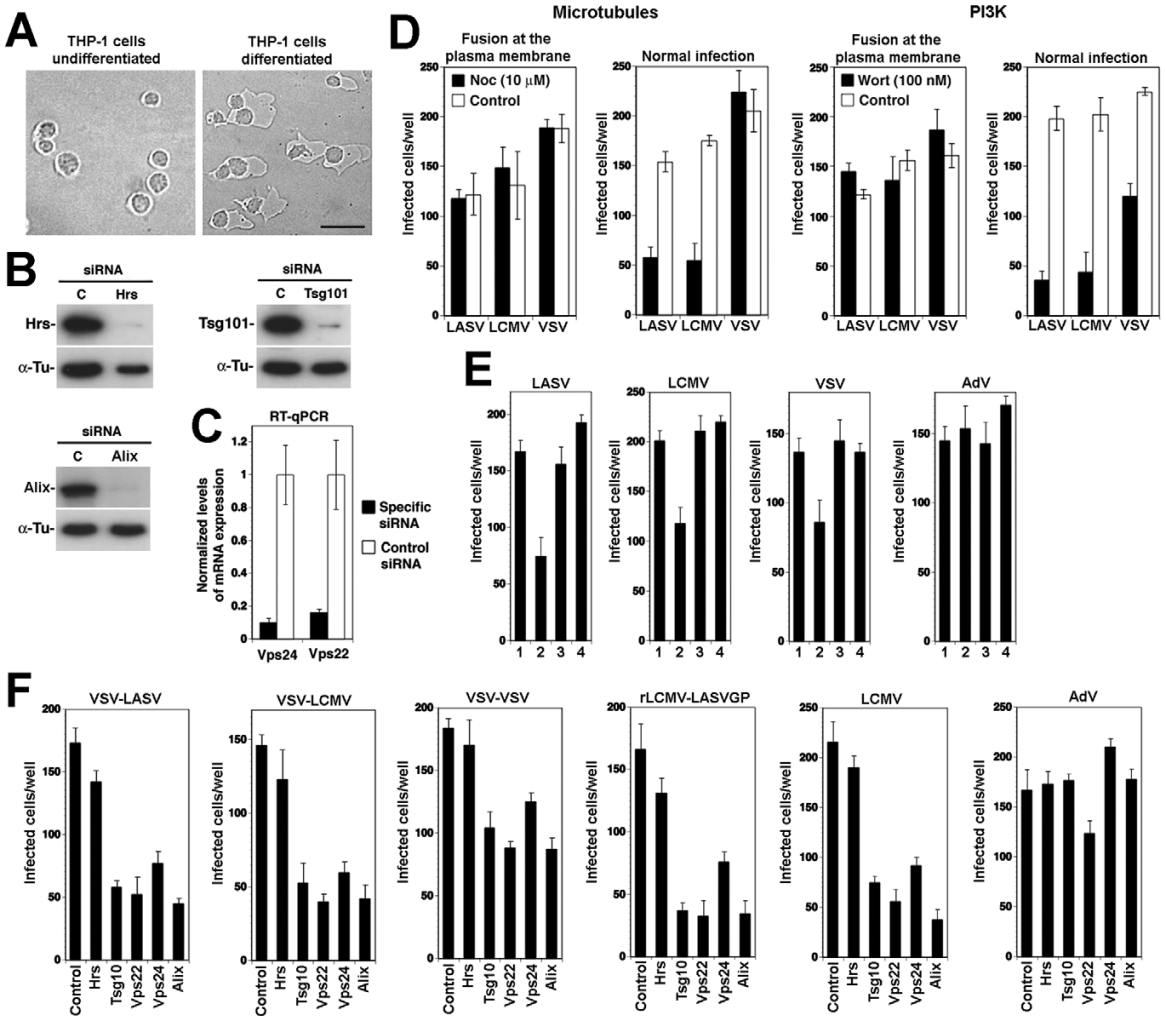
Cell entry of LASV and LCMV into human macrophages depends on the MVB and ESCRT proteins. (A) Differentiation of THP-1 cells into macrophages. THP-1 cells were treated with 50 ng/ml PMA for 48 hours and changes in cell morphology assessed by differential interference contrast microscopy (bar = 20 µM). (B) THP1 cells were seeded in 96 well plates (2×10^4^ cells/well) and differentiated into macrophage-like cells as in (A). Cells were then transfected with siRNAs for Hrs, Tsg101, Alix, or control siRNA and efficiency of depletion assessed after 48 hours by Western-blot as in Fig. 4A. For normalization, α-tubulin (α-Tu) was used. (C) Cells were transfected with siRNAs for Vps22 and Vps24 or control siRNA and efficiency of depletion assessed after 48 hours by quantification of mRNA levels by RT-qPCR as in Fig. 4B. (D) Cell entry of LASV and LCMV into human macrophage-like cells depends on PI3K and microtubules. THP1-derived macrophage-like cells generated as in (A) were treated with the indicated concentrations of wortmannin and nocodazole. Cells were then infected with rVSVΔG-LASVGP (LASV), rVSVΔG-LCMVGP (LCMV), and rVSVΔG-VSVG (VSV) either via fusion at the plasma membrane (2000 PFU/well) or via the normal route of infection (200 PFU/well) as in Fig. 2. Infection was detected by scoring EGFP positive cells (mean ± SD; n = 3). (E) Infection of VSV pseudotypes of LASV, LCMV, and VSV is perturbed by anti-LBPA treatment. THP-1-derived macrophage-like cells were pre-incubated with no antibody (1), 50 µg/ml mAb anti-LBPA (2) or isotype antibody control (3) for 14 hours. Cells were then infected with rVSVΔG-LASVGP (LASV), rVSVΔG-LCMVGP (LCMV), rVSVΔG-VSVG (VSV), and AdV5-EGFP at 300 PFU/well. In specimens subjected to pretreatment only (4), the cells were incubated for 1 h at 4°C with viruses in presence of the antibody, unbound virus washed out, and cell incubated at 37°C in normal medium. Cells were fixed after 16 hours and EGFP positive cells counted (mean ± SD; n = 3). (F) Cell entry of LASV and LCMV into human macrophages depends on Tsg101, Vps22, Vps24, and Alix, but not Hrs. THP-1-derived macrophage-like cells were subjected to RNAi silencing of Hrs, Tsg101, Vps22, Vps24, and Alix as in (B) and (C). After 48 hours cells were infected with rVSVΔG-LASVGP (VSV-LASV), rVSVΔG-LCMVGP (VSV-LCMV), rVSVΔG-VSVG (VSV-VSV), rLCMV-LASVGP, LCMV, and AdV5-EGFP (AdV) at 300 PFU/well. Infection was assessed after 16 hours by IFA (mean ± SD; n = 3).

Human cells of the monocyte/macrophage lineage are early and preferred targets of LASV in human and infection of monocytes and macrophages plays an important role in the pathogenesis of fatal Lassa fever [1], [2]. Considering their importance as targets *in vivo*, we investigated LASV cell entry into human cells of the monocyte/macrophage lineage and in particular the role of the MVB and ESCRT. For this purpose, we employed the human monocyte cell line THP-1, which once differentiated, represents a well-characterized model for human macrophages. When treated with phorbol 12-myristate 13-acetate (PMA) for 48 hours, the monocyte THP-1 cells undergo differentiation and adopt a macrophage-like phenotype with characteristic morphological changes [70] (Fig. 10A). In a first step, we optimized siRNA transfection conditions using the reagent HiPerFect and fluorescence-labeled siRNA and achieved transfection efficiencies of >90%. We then silenced Hrs, Tsg101, Vps22, Vps24, and Alix and assessed the efficiency of depletion after 48 hours by Western-blot and RT-qPCR. For all five targets, we obtained a degree of depletion similar to the one observed in A549 cells (Fig. 10B), making this cellular model suitable for our studies. To assess the role of PI3K and microtubules in LASV and LCMV cell entry into THP-1 derived macrophage-like cells, cells were treated with wortmannin and nocodazole, respectively, as described above. Cells were then infected with VSV pseudotypes of LASV, LCMV, and VSV via either fusion at the plasma membrane or normal cell entry. Similar to our results obtained in A549 cells (Fig. 2), normal cell entry of LASV and LCMV into macrophage-like cells was dependent on PI3K and microtubules (Fig. 10D). Next we studied the role of LBPA by feeding macrophage-like cells anti-LBPA antibody, prior to infection with VSV pseudotypes of LASV, LCMV, and VSV as well as AdV5-EGFP used as a negative control. As shown in Fig. 10E, pre-treatment with anti-LBPA, but not control IgG1 significantly reduced infection with pseudotypes of LASV, LCMV, and VSV, indicating a role of LBPA in LASV and LCMV entry into macrophage-like cells.

Lastly, we addressed the role of Hrs, Tsg101, Vps22, Vps24, and Alix in cell entry of LASV and LCMV into macrophage-like cells. Briefly, THP-1 cells were differentiated into macrophages and transfected with siRNAs to Hrs, Tsg101, Vps22, Vps24, and Alix. After 48 hours, cells were infected with VSV pseudotypes of LASV, LCMV, and VSV, rLCMV-LASVGP, LCMV, and AdV5-EGFP. Detection of infection after 16 hours revealed that depletion of Hrs had no effect on entry of any of the viruses, whereas silencing of Tsg101, Vps24, and Alix markedly reduced infection (Fig. 10F). Depletion of Vps22 affected infection with all viruses, but AdV5-EGFP to a lesser extent. Together, the data indicate an at least partially conserved cell entry pathway of LASV pseudotypes and LCMV between A549 cells and macrophage-like cells with roles for PI3K, microtubules, LBPA, the ESCT proteins Tsg101, Vps22, Vps24, and Alix, but distinct roles for the ESCRT-0 component Hrs.

## Discussion

In the present study we sought to identify cellular factors involved in the unusual pathway of cell entry used by Old World arenaviruses. We found that cell entry of LASV and LCMV required functional microtubules and PI3K activity. Productive viral infection depended on LPBA, a lipid involved in the formation of ILV of the MVB/late endosome, and components of the ESCRT complex. Productive infection with rLCMV-LASVGP and LCMV also critically depended on the ESCRT-associated protein Alix implicated in the membrane dynamics of late endosomes. In sum our study identifies crucial cellular components implicated in Old World arenavirus cell entry and indicates that these viruses invade the host cell passing through the MVB/late endosome using a pathway that may be involved in degradation of their cellular receptor.

Previous studies demonstrated a role for microtubules in early Old World arenavirus infection, but did not distinguish between a role in viral entry and a requirement for post-entry steps of replication [21]. In the present study we profited from the fact that VSV replication in the cytoplasm occurs independent of microtubules [23] and that recombinant VSV can be efficiently pseudotyped with the GPs of LCMV and LASV [71]. Using recombinant VSV pseudotypes, we found that cell entry of LASV and LCMV critically depended on microtubules prior to fusion, compatible with a role for microtubular transport in vesicular trafficking of the virus to the late endosome.

The unusual pathway of cell entry of LASV and LCMV is independent of known regulatory proteins associated with endocytosis such as clathrin, caveolin, dynamin, flotillin, or ARF6 and appears to bypass Rab5- and EEA1 positive early endosomes [19], [20], [21]. Infection of cells by LASV and LCMV critically depends on α-DG that functions as a high affinity receptor for Old World arenaviruses [14], [67], [72]. Considering the negligible off-rate of the virus-receptor binding [71], [73], it is conceivable that the virus-receptor complex is internalized and follows a pathway normally associated with endocytosis and degradation of α-DG. To test this hypothesis, we addressed the role of the MVB/late endosome in viral entry.

The formation of a functional MVB requires the biosynthesis of the membrane lipid PI3P by PI3K [41]. Since the post-fusion steps of VSV replication do not depend on PI3K activity [23], we again employed recombinant VSV pseudotypes to address a possible role for PI3K in cell entry of LASV and LCMV. In contrast to VSV that can undergo fusion at the level of the early endosome and is not inhibited by the PI3K inhibitor wortmannin [23], infection with the LASV and LCMV pseudotypes was markedly reduced. The dependence on PI3K at the level of viral entry provided a first hint towards a requirement for a functional MVB. To address the role of the MVB in LASV and LCMV cell entry more specifically, we perturbed the function of LBPA, an unusual lipid that is crucial for the formation of the MVB [42]. For this purpose we pre-treated cells with the well-characterized function-blocking antibody 6C4 to LBPA, which selectively prevents the formation of ILV in the MVB/late endosome [42]. Anti-LBPA treatment significantly perturbed LASV and LCMV cell entry, suggesting a role of the MVB. To address the role of the ESCRT complex, we depleted selected components of ESCRT-0 through III, namely Hrs, Tsg101, Vps22, and Vps24, respectively, using firmly established RNAi protocols. Depletion of Hrs, Tsg101, Vps22, and Vps24 under our experimental conditions markedly reduced cell entry of LASV and LCMV, but only mildly affected infection with AdV5 and did not affect early endosomal trafficking of TfR1, excluding general perturbation of membrane trafficking and/or endocytosis. Similar results were obtained upon over-expression of a DN variant of the ESCRT-associated ATPase Vps4 that is essential for the terminal fission of ILV. While depletion of Tsg101 markedly reduced viral infection, over-expression of recombinant Tsg101 significantly accelerated cell entry of LASV and LCMV, further supporting a role for Tsg101 as a positive regulator of Old World arenavirus entry. A role of the MVB for viral entry is also supported by the reduced susceptibility of cells depleted for Alix, an ESCRT-associated protein that is involved in the organization of the MVB in an LBPA-dependent manner [42]. To corroborate our findings obtained with RNAi and recombinant proteins that pinpoint a role for the ESCRT in Old World arenavirus entry, we performed co-localization studies between the prototypic LCMV and Tsg101 using confocal microscopy. Our studies indicate that incoming virus transiently passes through a compartment associated with Tsg101, and is subsequently delivered to Rab7 positive late endosomes, consistent with a passage through the MVB and sorting by the ESCRT.

The LCMV isolates used to study virus entry, LCMV WE54 [19] and LCMV cl-13 [20] bind to α-DG with high affinity and are dependent on α-DG for cell entry [67]. However, a single point mutation in the GP1 of WE54, S153F, present in the isolate WE22 [74], abolishes α-DG binding and makes WE22 independent of α-DG [67]. Comparison of the stability of WE54 and WE22 towards acidic pH revealed that WE22 retained the unusually high acid stability and low fusion pH found in LCMV WE54 and LASV [69]. Upon RNAi silencing of the ESCRT proteins Hrs, Tsg101, Vps22, and Vps24, as well as Alix, infection of the α-DG-independent LCMV WE22 was affected to a similar extent as the α-DG-dependent viruses LCMV WE54, LCMV cl-13, and rLCMV-LASVGP. Although by no means comprehensive, these studies with WE22 indicate that also an α-DG-independent LCMV isolate passes through the MVB and depends on ESCRT-mediated sorting for productive infection, which may be linked to the use of a yet unknown cellular membrane receptor and/or the unusually low fusion pH of the virus.

To study LASV and LCMV cell entry we largely used the human epithelial cell line A549. While a good model for epithelial cells that are important targets of arenaviruses *in vivo*, they are different from human cells of the monocyte/macrophage lineage, which represent key targets for LASV involved in the pathogenesis of fatal Lassa fever [2]. Using a well-characterized cell culture model for human macrophages, we assessed the roles of the MVB and ESCRT in LASV and LCMV cell entry. These studies revealed that LASV and LCMV cell entry into macrophage-like cells also depends on a functional MVB and the ESCRT components Tsg101, Vps22, and Vps24, as well as Alix. However, in contrast to A549 and HEK293 cells, infection of macrophage-like cells seemed independent of Hrs. Since Hrs is a constituent of ESCRT-0 that recruits cargo to the ESCRT-I, our data suggest that the initial events of LASV cell entry, prior to the interaction with ESCRT-I, are distinct in A549 cells and macrophages-like cells. This difference may be linked to differential receptor use and/or another endocytotic pathway involved in virus internalization. As befits their role in pathogen detection and antigen presentation, macrophages express a number of pathogen recognition receptors (PRR) and have pathways of endocytosis that do not exist in other cell types [75], [76]. Their role in LASV infection of human macrophages is currently unknown and under investigation in our laboratory.

The requirement for the MVB/late endosome for cell entry of LASV and LCMV found here is reminiscent to earlier reports on the cellular entry of the Anthrax toxin, the major virulence factor of *Bacillus anthracis* [60], [77], as well as studies on the endosomal transport of influenza virus [78] and VSV [23]. In case of Anthrax toxin and VSV, the pathogens undergo ESCRT-mediated sorting into ILV for transport to the late endosome, where penetration into the cytosol occurs by back-fusion of ILV with the endosomal membrane [23], [60]. Due to its pH optimum for fusion of >6.0, VSV penetrates the ILV membrane at an early step, resulting in the accumulation of the viral nucleocapsids in the lumen of ILV [23]. Delivery of VSV nucleocapsids into the cytoplasm at the level of the late endosome most likely occurs by back-fusion and involves LBPA, Alix, and Tsg101 [23], [79]. To “hide” the viral nucleocapsid in the lumen of ILV during the passage through the MVB/late endosome may be a strategy to protect the virus against the increasingly hostile biochemical environment of the late endosome. In contrast to VSV, LASV and LCMV have a remarkably low pH optimum for fusion (<5.0) [18], [69] and in particular LASV is unusually resistant to acidic pH [69]. It seems therefore rather unlikely that fusion can occur before the viruses reach late endosomal/lysosomal compartments.

**Figure 11:**
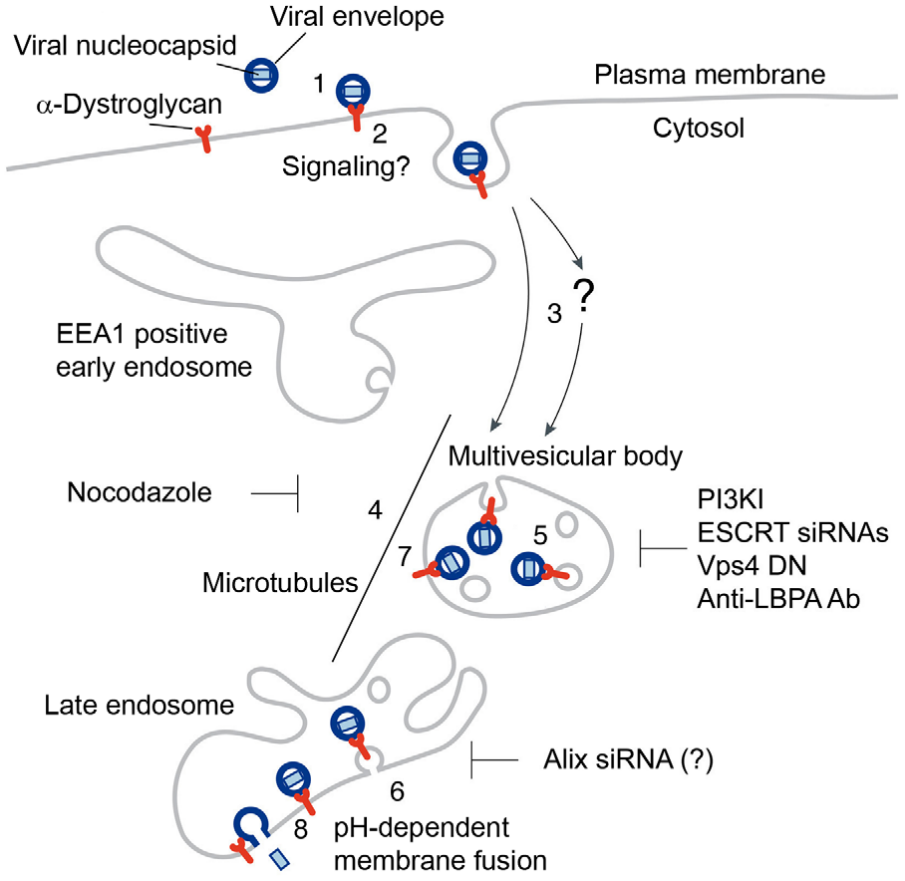
Working model for the cell entry of the Old World arenaviruses LASV and LCMV. For details please see text.

Based on previous studies and the present work, we propose a working model for the cell entry of Old World arenaviruses (Fig. 11). LASV, most LCMV isolates, and the related African arenaviruses Mobala and Mopeia use α-DG as a high affinity cellular receptor (1). Virus-receptor binding may result in receptor clustering, possibly accompanied by receptor-mediated signaling. Upon attachment, virus is rapidly internalized by smooth vesicles (2) [17], [19] involving a clathrin- and caveolin-independent pathway [19], [20], [21]. Based on the negligible off-rate of the virus binding to α-DG at neutral pH [71], [73], we assume that the virus-receptor complex is internalized. In a Rab5-independent manner, virus-containing vesicles may then be delivered either directly to the MVB (3) or passing through a yet unknown intracellular compartment (?), which seems distinct from classical EEA1 positive early endosomes. The MVB is a dynamic organelle characterized by the presence of ILV that gives raise to late endosomes. Delivery of the virus-receptor complex within the MVB to late endosomes seems to depend on microtubular transport (4), but independent of Rab7 [19], [21]. At the level of the MVB, the virus-receptor complex may undergo ESCRT-mediated sorting into ILV (5), followed by delivery to the late endosome. To allow penetration of the viral nucleocapsid into the cytosol, ILV bearing virus-receptor complexes would have to undergo back-fusion at the level of the late endosome, which may depend on Alix and LBPA (6). However, we cannot exclude the possibility that the virus-receptor complex may to some extent remain associated with the limiting membrane of the MVB (7) and fusion may be initiated as the intraluminal pH progressively drops (8).

A hallmark of fatal LASV infection in humans is the inability of the host cell's innate immune system to detect and contain the virus, resulting in uncontrolled infection [2]. Instead of being recognized as a foreign antigen, LASV escapes innate pathogen detection and establish a productive infection without inducing an interferon response [80], [81]. An important class of cellular PRRs that allows early detection of incoming RNA viruses are transmembrane receptors of the Toll-like family localized in the Rab5/EEA1 positive early endosome [82]. The ability of LASV to use a pathway of endocytosis bypassing classical routes of incoming endosomal trafficking followed direct delivery to late endosomes may contribute to LASV's ability to escape detection by endosomal receptors of innate anti-viral defense, a hypothesis we are currently testing.

## Materials and Methods

### Antibodies and reagents

Mouse monoclonal antibody (mAb) 113 anti-LCMV NP has been described previously [83]. Mouse mAbs recognizing Hrs and Tsg101 were purchased from Alexis Biochemicals (Lausen, Switzerland) and GeneTex (Irvine, CA), while rabbit polyclonal antibody (pAb) anti-Alix was from Covalab (Villeurbanne, France). MAb IIH6 anti-α-DG has been provided by Dr. Kevin Campbell (Howard Hughes Medical Institute, University of Iowa). MAb to clathrin heavy chain was purchased from BD Bioscience (San Jose, CA). Mouse mAb anti-FLAG M2, rabbit pAb anti-FLAG and mouse mAb anti-α-tubulin were all obtained from Sigma-Aldrich (St. Louis, MO). Mouse mAb 6C4 anti-LBPA [84] was a kind gift of Dr. Jean Gruenberg (University of Geneva, Switzerland). Mouse mAb 23H12 specific for the M protein of VSV [85] was kindly provided by Dr. Douglas S. Lyles (Wake Forest University School of Medicine, NC). Mouse mAb anti-MYC was produced from the 9E10 hybridoma. Horseradish peroxidase (HRP) conjugated secondary antibodies were from Pierce (Rockford, IL), rhodamine red-X goat anti-mouse IgG from Jackson ImmunoResearch (Suffolk, UK), while Alexa 594 goat anti-mouse IgM, Alexa 488 goat anti-mouse IgG_2a_, Alexa 647 goat anti-rabbit IgG and 4′,6-diamidino-2-phenylindole (DAPI) were purchased from Molecular Probes (Eugene, OR) and phorbol 12-myristate 13-acetate (PMA) was obtained from Sigma.

### Cells and viruses

Human lung adenocarcinoma epithelial cells (A549), human embryonic kidney cells (HEK293), African green monkey kidney cells (Vero E6), North African green monkey (*Cercopithecus aethiops*) kidney fibroblasts (CV1), and Syrian golden hamster kidney cells (BHK-21) were maintained in Dulbecco's modified Eagle medium (DMEM) (Gibco BRL, NY) containing 10% FBS. THP-1 cells were maintained and differentiated into macrophages as described [70]. LCMV clone 13 (cl-13) and recombinant LCMV carrying the LASV GP (rLCMV-LASVGP) have been already described [21], [86]. Seed stocks of LCMV were prepared by growth in BHK-21 cells, and titers were determined as reported previously [87]. Wild-type vesicular stomatitis virus (VSV) (Indiana serotype) was grown in Vero E6 cells, and titers were determined by plaque assay on Vero E6 cells. Recombinant VSV pseudotyped with LASV GP (rVSVΔG-LASVGP), LCMV GP (rVSVΔG-LCMVGP), and VSV GP (rVSVΔG -VSVG) were generated as reported previously [71]. Virus titers were determined by the infection of Vero E6 cell monolayers and detection of GFP-positive cells by fluorescence microscopy.

### Virus infection and fusion assay

For virus infection, 2×10^4^ cells per well were seeded in 96 well plates and cultured overnight. For infection of cells with LCMV, rLCMV-LASVGP and VSV, seed stocks were diluted to the indicated MOI and added to cells for 1 h at 37°C. After 1 h of incubation, the inoculum was removed, and replaced with normal medium. To prevent secondary infection, 20 mM NH_4_Cl was added to the cells 4 h after infection. Cells were fixed 5 (VSV) or 16 (LCMV cl-13 and rLCMV-LASVGP) h after infection and infected cells quantified by immunofluorescence assay (IFA) detection of LCMVNP and VSVM using mAbs 113 (anti-LCMVNP) and 23H12 (anti-VSVM) combined with fluorescence-labeled secondary antibodies as in [88]. Images of infected cells within one experiment were acquired with the same microscope settings using a Zeiss LSM 510 Meta confocal microscope (Zeiss) and a 40×, 1.3 NA or a 20× objective.

For infection of cells with rVSVΔG-LCMVGP, rVSVΔG-LASVGP and rVSVΔG-VSVGP, seed stocks were diluted to the indicated MOI and added to cells for 1 h at 37°C. After 1 h of incubation, the inoculum was removed, and replaced with normal medium. Cells were fixed at 12 h after infection and GFP-positive cells, or cells stained with mAb 23H12 to VSV-M, scored as infected by flow cytometry or fluorescence microscopy.

The kinetics of cell entry by ammonium chloride treatment was performed as described [21]. Briefly, cells were cooled on ice for 30 min and viruses added the indicated MOI. After incubation for 1 h on ice, unbound virus was washed off and cells were quickly shifted to 37°C. After the indicated time points, 20 mM ammonium chloride was added to the medium and kept throughout the experiment. After a total of 12 (rVSVΔG-LASVGP and rVSVΔG-VSVGP) or 16 (rLCMV-LASVGP) h, cells were fixed, stained and analyzed by flow cytometry.

### Pharmacological inhibitors

For cholesterol extraction with methyl-β-cyclodextrin (MβCD), medium was removed, cells washed twice with medium without FBS, and MβCD added at the indicated concentrations for one hour at 37°C, 5% CO_2_. Cells were washed three times with medium to remove residual MβCD and infection assays performed as described. For the quantitative determination of cellular cholesterol, cells were extracted with chloroform and methanol (2∶1) and cholesterol quantified by a commercial colorimetric assay (BioVision Inc. Mountain View, CA) according to the manufacturer's recommendations. Treatment with chlorpromazine (CPZ) was performed as described [28]. Studies with nocodazole to address the role of microtubules and wortmannin to define the role PI3K for cell entry of VSV pseudotypes were performed as described [23]. All pharmacological inhibitors were purchased from Sigma-Aldrich (St. Louis, MO).

### Infection assays with wild-type and dominant negative mutants of dynamin, Eps15, and Rab5

Green fluorescent protein (GFP)-tagged wild-type and DN (K44E) dynamin II [38] were kindly provided by Dr. Sandra L. Schmid (The Scripps Research Institute, CA) and the control Eps 15DIIIΔ2 construct [34] as well as the DN Eps15Δ95/295 mutant construct [89] were provided by Drs Alice Daurty-Varsat and Nathalie Sauvonnet (Institut Pasteur, Paris). Constructs of wild-type GFP-tagged human Rab5A and the constitutively inactive mutant Rab5 S34N [90], [91] were provided by Dr. Craig Roy (Yale University School of Medicine, New Haven, CT). The impact of transgene expression on virus infection was assessed as described [20]. Briefly, cells were transiently transfected with plasmid DNA using the Nucleofector system (Amaxa, Gaithersburg, MD) according to the manufacturer's protocols. Transfection efficiencies with plasmids, as assessed by detection of GFP, were >90% for HEK293 cells. Cells expressing GFP-tagged constructs for 20 hours were infected with VSV pseudotypes rVSVΔG-LASVGP, rVSVΔG-LCMVGP and rVSVΔG-VSVG at 200 PFU/well. Infection of VSV pseudotypes was detected by IFA using mAb 23H12 specific for the M protein of VSV and a rhodamine red X-conjugates secondary antibody. The number of VSV M positive cells was determined for each well in triplicates.

### RNA interference (RNAi)

The depletion of clathrin heavy chain by RNAi was performed as described in [92] using ON-TARGETplus SMARTpool and a control siRNA pool obtained from Dharmacon (Lafayette, CO). Briefly, cells (2×10^4^ cell/well) were seeded in 96 well plates and transfected with siRNAs at concentrations of 50 nM per siRNA, corresponding to a total concentration of 4×50 = 200 nM, using Lipofectamine 2000. After 48 hours, cells were lysed and depletion of clathrin heavy chain and caveolin-1 detected by Western-blot analysis, using α-tubulin for normalization.

For the knockdown of ESCRT components, validated siRNAs for human Hrs (SI00288239), Alix (SI02655345) and scrambled siRNA (1027280) were purchased from Qiagen (Basel, Switzerland) while target sequences for human Tsg101, Vps22 and Vps24 were previously described in [93], [50], and [49] respectively. In RNAi experiments, A549, HEK293, and CV-1 cells were transfected twice at a 24 h interval with 16 nM siRNA using Lipofectamine RNAiMAX (Invitrogen, Paisley, UK) according to the manufacturer's instructions, replated the day after the second transfection and further manipulated 24 h later. THP-1 cells were differentiated by the addition of PMA (50 ng/ml) for 48 hours and then transfected with HiPerFect (Qiagen) according to the manufacturers recommendation, using 50 nM of siRNA.

### Anti-LBPA antibody treatment

BHK-21 cells were left untreated, pre-incubated with 50 µg/ml of anti-LBPA antibody or with 50 µg/ml of isotype IgG control for 14 h and infected with LCMV, rLCMV-LASVGP and VSV. To exclude a direct effect of the anti-LBPA Ab on virus infectivity, untreated cells were incubated for 1 h at 4°C with LCMV, rLCMV-LASVGP and VSV in presence of the Ab, unbound virus washed out, and cell incubated at 37°C in normal medium.

### Immunoblotting

Standard immunoblotting involved proteins being separated by SDS-PAGE gel electrophoresis and transferred to nitrocellulose. After blocking in 3% (wt/vol) skim milk in PBS, membranes were incubated with 1–10 µg/ml primary antibody in 3% (wt/vol) skim milk, PBS overnight at 4°C. After several washes in PBS, 0.1% (wt/vol) Tween-20 (PBST), secondary antibodies coupled to HRP were applied 1∶5,000 in PBST for 1 h at room temperature. Blots were developed by enhanced chemiluminescence (ECL) using Super Signal West Pico ECL Substrate (Pierce).

### Real time PCR

Total RNA was purified with RNeasy Mini Kit (Qiagen) and cDNA synthesized using QuantiTect Reverse Transcription Kit (Qiagen). TaqMan probes specific for Vps22 (Hs00273125_m1), Vps24 (Hs00984915_m1) and PRKCSH (Hs00160457_m1) were obtained from Applied Biosystems. Real Time PCR was performed using StepOne Real-Time PCR System (Applied Biosystems) and gene expression levels relative to PRKCSH determined according to the 2^−ΔΔ*C*T^ method [94].

### Transferrin uptake assay

Transferrin uptake assay was performed according to [95]. Cells were incubated for 10 min at 37°C with serum-free medium containing 20 µg/ml of Alexa594-labeled human transferrin (Invitrogen), washed with ice cold PBS and acid-stripped (150 mM NaCl, 2 mM CaCl_2_, 25 mM CH_3_COONa, pH 4.5) to remove surface-bound transferrin. Cells were immediately fixed with 2% formaldehyde in PBS for 15 min and specimens examined with the same microscope settings using a Zeiss LSM 510 Meta confocal microscope (Zeiss) and a 40×, 1.3 NA objective.

### Infection assays with wild-type and dominant negative mutants of Vps4A

Expression plasmids for human FLAG-tagged Vps4A and Vps4A mutant E228Q (Vps4AEQ) [56] were kindly provided by J. Yasuda (Japan Science and Technology Agency, Japan). HEK293 cells were transfected using FuGENE6 and 36 h after cells infected with LCMV or rLCMV-LASVGP (MOI∼1). 20 mM ammonium chloride was added 4 hours post infection to prevent secondary infection. Sixteen h post infection, cells were fixed and immunostaining against LVMV-NP and FLAG tag performed. Two populations of cells were selected in each sample: a “non-expressing” population negative for flag and an “expressing” population containing the construct. The percentage of cells infected within each population was quantified in a histogram of the virus staining intensity. The effect of a given flag-fusion protein was expressed as normalized infection level, i.e., the ratio of the infection level in the cell population expressing the protein divided by the infection level in the non-expressing cell population.

### Tsg101 overexpression

pC-Tsg101 [96] carrying a MYC tag upstream the human coding sequence was from S. N. Cohen (Stanford University School of Medicine, CA). In transfection experiments, HEK293 cells were transfected using FuGENE6 (Roche, Basel, Switzerland) according to the manufacturer's protocol, and infected 36 h after.

### Cell surface staining of α-DG

For cell surface staining, cells were detached with enzyme-free cell dissociation solution (Sigma-Aldrich), resuspended in FACS buffer (1% (vol/vol) FBS, 0.1% (wt/vol) sodium azide, PBS), and plated in conical 96-well trays. For cell surface staining of functionally glycosylated α-DG, cells were incubated with mAb IIH6 (1∶100). Incubation was for one hour on ice in FACS buffer. Cells were then washed twice in FACS buffer and labeled with Alexa 594-conjugated secondary antibodies (1∶100 in FACS buffer) for 45 min on ice in the dark. After two wash-steps in 1% (vol/vol) FBS in PBS, cells were fixed with 4% (wt/vol) paraformaldehyde, PBS for 10 min at room temperature in the dark. The cells were washed twice with PBS, and analyzed with a FACSCalibur flow cytometer (Becton Dickinson, San Jose CA) using Cell Quest software.

### Virus inactivation assay

To compare the fusion pH of different LCMV isolates, viruses were incubated with 100 mM sodium citrate solutions buffered at the indicated pH for 15 min. Titers after incubation were determined by infection of A549 monolayer as described previously [68].

### Co-localization analysis

To perform co-localization studies A549 cells previously seeded on glass 8-well LabTeks were cooled on ice for 30 min, incubated for 1 h on ice with LCMV WE54 (MOI∼100) to allow virus binding and then washed with cold medium. Cells were then shifted at 37°C and fixed at the indicated time points with 2% formaldehyde in PBS for 15 minutes at RT and washed with PBS. Cells were permeabilized for 30 min at RT with 0.1% saponin, 10% goat serum, 100 mM glycine in PBS. Primary (mouse monoclonal anti LCMV NP clone 113 2 µg/ml, rabbit polyclonal anti Tsg101 1∶100, GeneTex, and rabbit polyclonal anti-Rab7 1∶100, Cell Signaling) and secondary antibodies (Alexa488-F(ab′)_2_ fragment of goat anti-mouse IgG and Alexa595-F(ab′)_2_ fragment of goat anti-rabbit IgG, Molecular Probes) were diluted in PBS, 0.1% saponin, 1% goat serum and incubated o/n at 4°C and 1 h at RT, respectively. Before acquisition nuclei were stained with 300 nM DAPI and LabTek were mounted with ProLong Gold (Molecular Probes). Image acquisition was performed with a Zeiss LSM710 Quasar confocal microscope equipped with a plan apochromat 63×, 1.2 NA objective and a 405 nm diode laser, a 458-476-488-514 nm Argon laser and a 561 nm DPSS. All images for each data set were acquired the same day with the same microscope settings. Images were first deconvolved using Huygens Essential (SVI, Hilversum, Netherlands) and then analyzed for colocalization with Imaris 7.2 (Bitplane). For each image a colocalization channel was built thresholding the scatter plot. Total number of viruses and the number of viruses in the colocalization channel were automatically quantified by image segmentation using Imaris spot detection function to model point-like structures. Ten randomly selected cells for each time points were analyzed.
