# Correction: Old World Arenaviruses Enter the Host Cell via the Multivesicular Body and Depend on the Endosomal Sorting Complex Required for Transport

**DOI:** 10.1371/annotation/952387a7-96a0-44f6-98a6-2d7c7b472b0a

**Published:** 2011-09-14

**Authors:** Giulia Pasqual, Jillian M. Rojek, Mark Masin, Jean-Yves Chatton, Stefan Kunz

The following information was missing from the funding section: This work was supported by Swiss National Science Foundation grants Nr. 3100AO-120250 and Nr. 310030-132844.

